# Modeling and analyzing cross-transmission dynamics of related information co-propagation

**DOI:** 10.1038/s41598-020-79503-8

**Published:** 2021-01-11

**Authors:** Fulian Yin, Xueying Shao, Biao Tang, Xinyu Xia, Jianhong Wu

**Affiliations:** 1 Communication University of China, Beijing, 100024 China; 2 York University, Toronto, M3J1P3 Canada

**Keywords:** Applied mathematics, Information technology

## Abstract

The dissemination of one public hot event is usually affected by some related information, and the implication of co-propagation by different information is critical for the integrated analysis. To help in designing effective communication strategies during the whole event, we propose the cross-transmission susceptible-forwarding-immune (CT-SFI) model to describe the dynamics of co-propagation particularly with focus on the cross-transmission effects. This model is based on the forwarding quantity and takes into account the behavior that users may have a strong attraction or continuous attraction within or without an active time after contacting one information. Data fitting using the real data of Chinese Sina-microblog can accurately parameterize the model and parameter sensitivity analysis gives some strategies for co-propagation.

## Introduction

One public hot event on the internet usually consists of multiple information^1^ and the mutual influence of them plays a significant role in the integrated propagation. For example, during the American election in 2016, Dan Scavino (https://weibo.com/5603237555/DizsYpU1p?sudaref=www.baidu.com&display=0&retcode=6102&type=comment#_rnd1578554984793) and Clint Eastwood (http://www.sohu.com/a/118499160_109901) indicated their support to Trump posted on Twitter, which helped Trump gain more supporters and promoted the spread of the election. And in 2019, the fake news about Hong Kong police released by BBC and Guardian (http://world.chinadaily.com.cn/a/201908/08/WS5d4be475a31099ab995d8363.html) received a lot of reposts, which caused the spreading of rumors and had serious effects on the police’s reputation. Modeling the dynamics of multiple information is an important issue for the complicated public opinion ecosystem^2^ and serves as a basis for complex dissemination.

Traditionally, researches on information dissemination always focus on single information, and a series of studies were carried out on the spread of rumors especially. Considering that rumors spread in a similar way to the epidemiology, many scholars used susceptible-infected (SI) model^3,4^, susceptible-infected-recovered (SIR) model^5,6^ and susceptible-exposed-infected-recovered (SEIR) model^7,8^ to represent rumor propagation. Then, some following researches improved the models to be more targeted and effective. On one hand, some scholars considered different states of the propagation and introduced distinct modules. In 2012, Zhao et al.^9^ developed a new rumor spreading model called susceptible-infected-hibernator-removed (SIHR) model and introduced a new kind of people-Hibernators to reduce the maximum rumor influence. In 2014, Zhao et al.^10^ proposed a SIR model and analyzed the dynamic process of rumor propagation by accounting for the refutation mechanism in homogeneous social networks, which could help authorities reduce the maximum influence of the rumor. Chen et al.^11^ studied the effect of the nodes’ role in the network on rumor’s suppression. Trpevski D et al.^12^, Qian et al.^13^, Wang et al.^14^ also proposed many improved models for the spread of rumors. On the other hand, some scholars incorporated more factors to represent the complex real world. In 2015, Zhang et al.^15^ studied the cumulative effects of memory on rumor spreading by using the data set of Chinese Sina-Microblog, and proposed a rumor spreading model which examined how the memory affected rate changes over time in the artificial network and the real social network. Zhang et al.^16^ developed the dynamic 8-state ignorance-carrier-spreader-advocate-removal (ICSAR) rumor propagation model to study the mechanism of rumor propagation and then studied the function of each influencing factor, which could improve the efficiency of rumor refutation and help to make emergency plans. Huang et al.^17^ constructed a model that considered the impact of rumor refuting by the affected enterprise, a microblogging opinion leader and a microblogging platform. Moreover, some studies also mentioned other factors^18–20^, such as the degree of trust, the strength of ties and the social intimacy degree between people.

In addition to rumors, some works studied the spread of general information. Liu et al.^21^ built a susceptible-antidotal-infected-removed (SAIR) model based on well-known epidemic models to characterize super-spreading phenomenon in tweets information propagation accompanied with super-spreaders, which was much more promising than the conventional SIR model in characterizing a super-spreading event of information propagation. Zhang et al.^22^ analyzed Sina-Microblog’s topological features, and brought in an epidemiological susceptible-exposed-infected-resistant (SEIR) model to explore the mode of message spreading throughout the microblog network. This work indicated that the network was small-world and scale-free, which made it succeed in transferring messages but failed in resisting negative influence.

There are many researches on cross-transmission in the field of disease spreading. For example, Tang et al.^23^ noticed the phenomenon of Zika virus co-propagation with dengue in tropical and sub-tropical regions and then formulated a model to describe the transmission dynamics of co-infection of these viruses. Different from Tang’s theoretical basis, Feng et al.^24^ developed a mathematical model that incorporated the virus mutation dynamics in the transmission of CHIKV among mosquitoes and humans. Meanwhile, the co-propagation phenomenon between the disease and the disease-related information had already described in complex networks^25^. Zhan et al.^26^ built a mathematical model based on the susceptible-infected-susceptible (SIS) process to investigate the interaction between the propagation of H7N9 and information about this disease, which described both spreading dynamics to illustrate the influence of information propagatiodemic spreading. In 2018^27^, analyzed the propagation and related information of two representative diseases named H7N9 and Dengue fever, then proposed a nonlinear model to further interpret the coupling effect. In addition^28^, proposed an information-driven adaptive model in which disease and disease information could evolve simultaneously, and simulation results indicated if susceptible individuals were able to recognize the disease then the information-driven adaptivity could slow down the speed of epidemic spreading and diminish the epidemic prevalence at the final state.

And in the field of spreading dynamics, there was still an example of co-propagation. In 2018, Zan et al.^2^ studied the double rumors spreading with different launch times and introduced two models: double-susceptible-infected-recovered (DSIR) model and comprehensive-DSIR (C-DSIR) model, which focused on the interaction from an old rumor to a new rumor. Then they provided the double-rumors dissemination mechanism by states-vectors expressions and introduced a selection parameter to express the attractions of different rumors. To the best of our knowledge, their paper firstly investigated the propagation of two rumors posted successively, which is the most related study to our works. However, they only considered the influence of the first rumor on the later one and ignored the interaction between two related information. Our paper will solve this limit, and it is the most important issue that we focus on in our paper.

**Figure 1:**
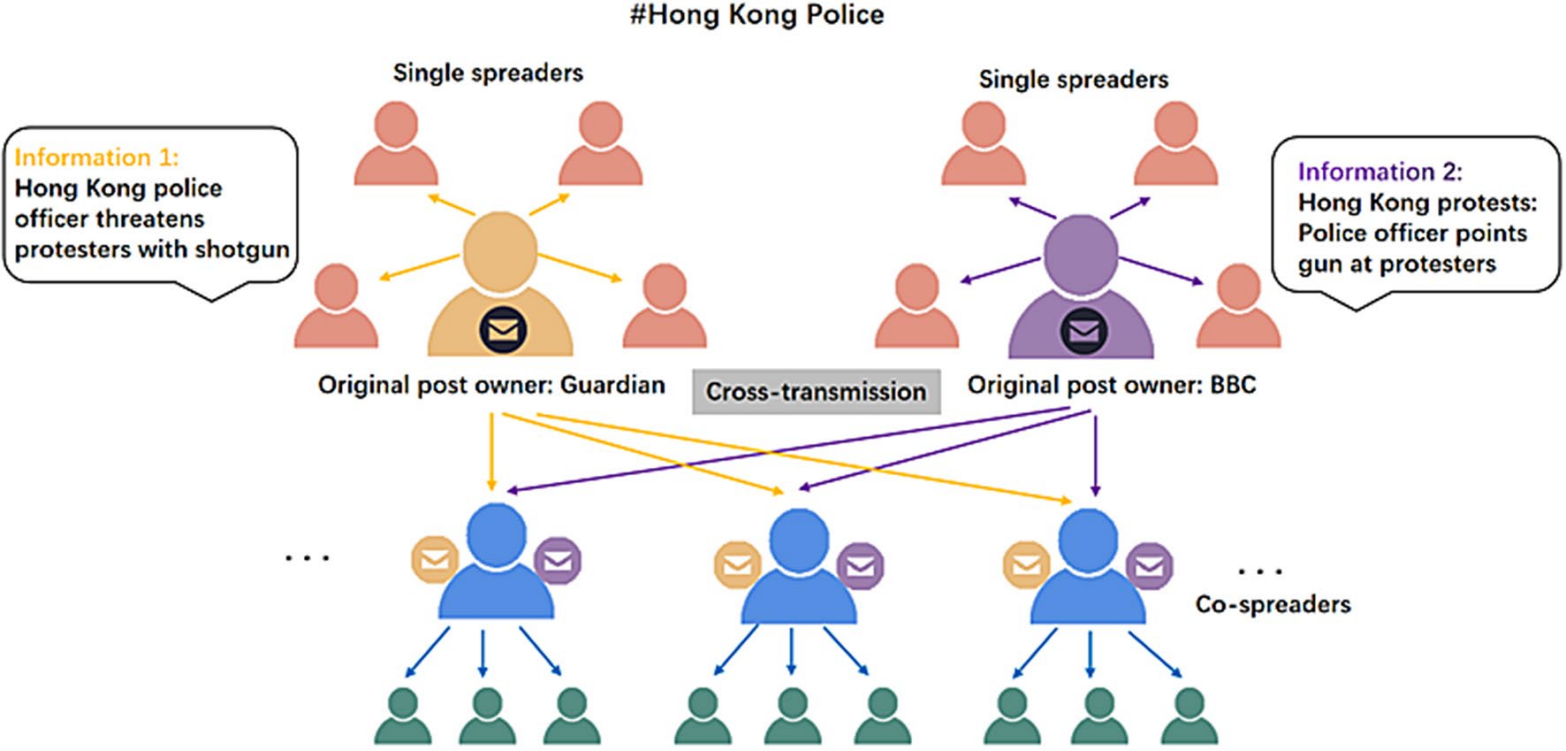
Cross-transmission of one public hot event about Hong Kong police.

Figure 1 shows the cross-transmission of one public hot event about Hong Kong police. As two original post owners, Guardian and BBC published negative information about Hong Kong police on twitter. This information received a lot of attention and led to the spread of the whole event. Especially, some users (co-spreaders) forwarded the second information because of the attraction of contacting the first information. In general, the cross-transmission can give rise to users’ interest in public hot events and promote information dissemination rapidly.

We propose a cross-transmission susceptible-forwarding-immune (CT-SFI) model, which takes into account the dynamics of interaction propagation based on the standard susceptible-forwarding-immune (SFI)^29^ process. Our model can be arguably considered as a variant of classic models in diseases area^23,24^, but the difference is that the dynamics of our model include the mutual forwarding with strong attraction and the mutual forwarding with continuous attraction. In the former process, a user will forward another information with strong interest after having forwarded one information within a short time, so we propose a strong attractive index, which describes the attraction from information *i* to information *j*, to learn the state transition in an active forwarding period. In addition, since the active state of the user is timeliness, we distinguish the population of cross-transmission from time. The latter process, a user who has contacted one information is still interested in the related information after a while, we propose a continuous attractive index, which portrays the attraction of state transition from information *i* to information *j*, to learn the state transition within an insensitive period of information *i*. Finally, we build a set of propagation indices and use numerical simulations on a real-world dataset to explore the interaction of CT-SFI for double information spreading. The results show that our model can effectively track and control the development trends of two related information in the design of priority strategies.

The rest of this paper is organized as follows: the mathematical model definition for cross-transmission is introduced in “Preliminaries”; the propagation pattern of the model is shown in "Model formulation"; in "Co-propagation indices", we establish an indices system of the model and calculate it; in "Data fitting", we fit the model with the real data from Chinese Sina-Microblog; in “Cross-transmission analysis”, we analyze the cross-transmission by two related information; in “Co-propagation analysis”, we analyze the integrated co-propagation further; in “Information co-propagation control strategy”, we give effective strategy about the information co-propagation. Our data fitting results show that the cross-transmission has an important impact on co-propagation between two information.

## Preliminaries

Co-propagation with cross-transmission by two related information is a normal behavior, here we make an assumption: users who contact one information may have a special interest in the other information as well, and keep susceptible in that.

**Figure 2:**
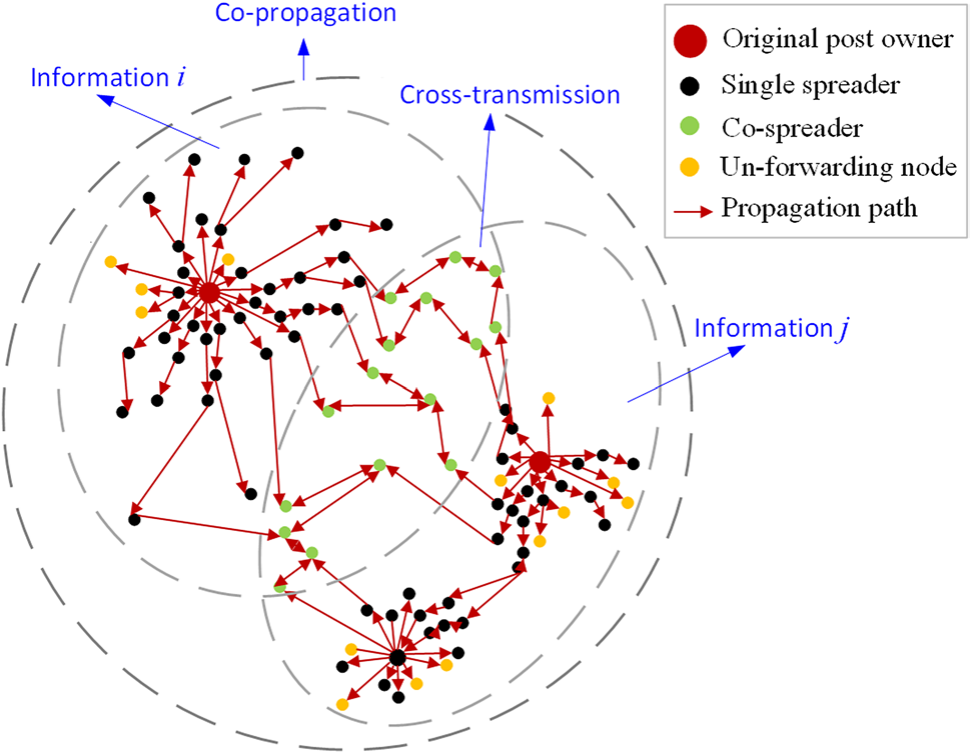
A schematic diagram for co-propagation by two relative information.

A schematic diagram is shown in Fig. 2: at the beginning of the public hot event, two post owners are the original spreaders of the information (information *i* and information *j*, *i*=1 or 2, *j*=1 or 2, and $i\not =j$, such as big red nodes), then, the information is forwarded separately by some single spreaders who are interested in them, such as the black nodes. Especially, some co-spreaders forward the information they contacted later due to the influence of the first information they contacted before, such as the green nodes. We call the process of second forwarding as cross-transmission and the whole process of information dissemination as co-propagation.

**Figure 3:**
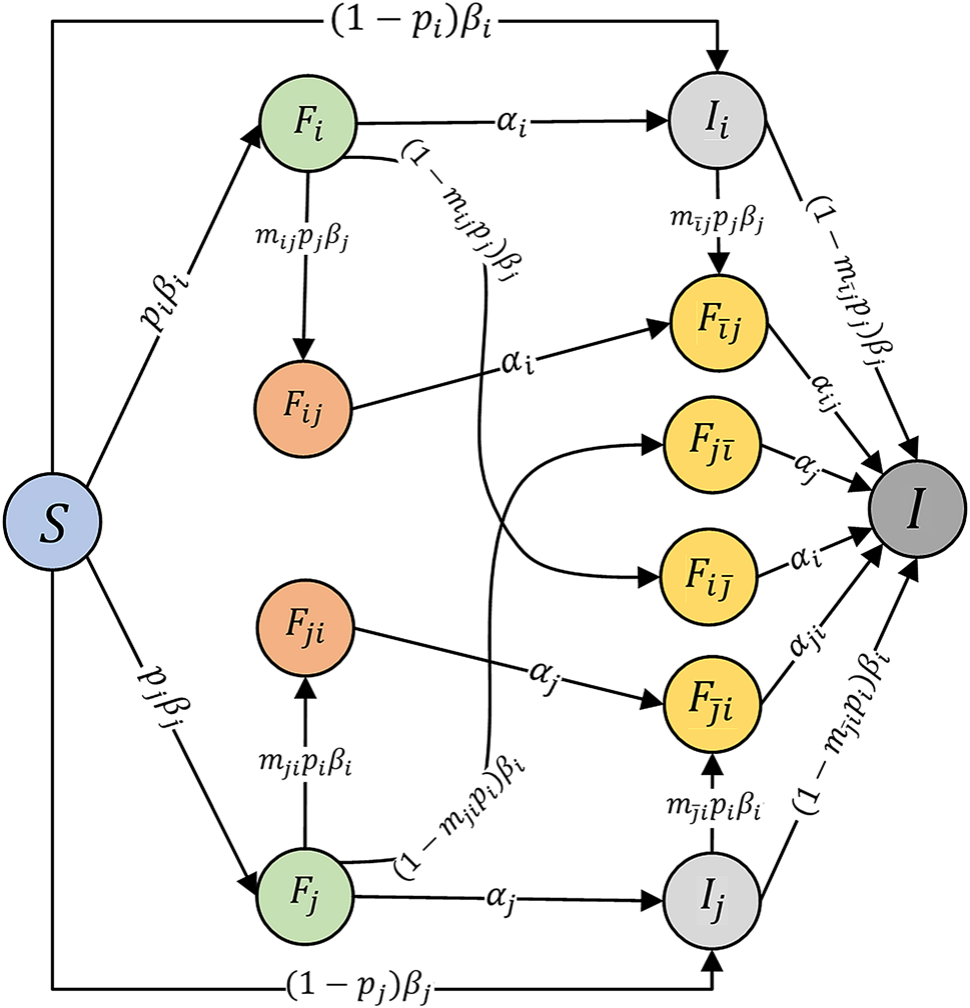
Cross-transmission flow chart for co-propagation.

Our cross-transmission susceptible-forwarding-immunized model (CT-SFI) is illustrated in the flow chart of Fig. 3. At time *t*, a single user *x* has two states for information *i* and information *j* respectively, which can be expressed as $$\left( \begin{array}{c} state\ of\ information\ i\\ state\ of\ information\ j\\ \end{array} \right) _x$$ and each state can be described as three distinct types: the susceptible state (*S*), in which users are unaware of but susceptible to the information; the forwarding state (*F*), in which users have been forwarding the information actively to influence other users, because the information cannot be contacted once out of an active forwarding period; and the immune state (*I*), in which users consist of two groups, one group is who have already forwarded the information but are no longer forwarding that even if receiving it again, which means they are out of an active forwarding period, and the other group is who get direct immunity after exposure to the information because they subjectively do not want to forward the information.

Considering the whole process of cross-transmission for co-propagation, we stratify the user population (*N*) into:

- *S*(*t*): at time *t*, the number of susceptible users unaware of but susceptible to both information.
- $F_{i} (t)$: at time *t*, the number of forwarding users to information *i* who have been only forwarding information *i* and actively to influence other users, also, susceptible to information *j*.
- $F_{j} (t)$: at time *t*, the number of forwarding users to information *j* who have been only forwarding information *j* and actively to influence other users, also, susceptible to information *i*.
- $I_i (t)$: at time *t*, the number of immune users to information *i* who have already forwarded the information *i* and after a while out of an active forwarding period; another kind who get direct immunity from the susceptible users through exposure to the information *i* differently. Also, they are susceptible to the other information *j*.
- $I_j (t)$: at time *t*, the number of immune users to information *j* who have already forwarded the information *j* and after a while out of an active forwarding period; another kind who get direct immunity from the susceptible users through exposure to the information *j* differently. Also, they are susceptible to the other information *i*.
- $F_{ij} (t)$: at time *t*, the number of forwarding users to both information who have been forwarding information *i* then information *j* successively, and both of the forwarding are in an active period to influence other users.
- $F_{ji} (t)$: at time *t*, the number of forwarding users to both information who have been forwarding information *j* then information *i* successively, and both of the forwarding are in an active period to influence other users.
- $F_{\bar{i} j} (t)$: at time *t*, the number of users immune to information *i* and in an active forwarding period of information *j*. Especially, the immune state of information *i* precedes the active forwarding state of information *j*.
- $F_{\bar{j} i}(t)$: at time *t*, the number of users immune to information *j* and in an active forwarding period of information *i*. Especially, the immune state of information *j* precedes the active forwarding state of information *i*.
- $F_{{i} \bar{j}}(t)$: at time *t*, the number of users in an active forwarding period of information *i* and immune to information *j*. Especially, the immune state of information *j* occurs later than the active forwarding state of information *i*.
- $F_{{j} \bar{i}}(t)$: at time *t*, the number of users in an active forwarding period of information *j* and immune to information *i*. Especially, the immune state of information *i* occurs later than the active forwarding state of information *j*.
- *I*(*t*): at time *t*, the number of users immune to both information.

The definition for parameters is listed in Table 1, where $\beta$ and $\alpha$ are traditional parameters in standard epidemic-SIR models and *p* is a parameter reflecting the probability that the exposed user will forward the information in a standard susceptible-forwarding-immune (SFI) model^29^ for information propagation. In addition, some other important parameters in our model to measure the degree of cross-transmission are $m_{ij}$ $(m_{ji})$ and $m_{\bar{i}j}$ $(m_{\bar{j}i})$, which reflect the attraction of two related information. Here, $m_{ij}$ $(m_{ji})$ denotes the degree of strong attraction, within the active period of forwarding the first information, to forward the second information. And $m_{\bar{i}j}$ $(m_{\bar{j}i})$ denotes the degree of continuous attraction, within an insensitive period of first information, to forward the second information due to the influence of contacting the first information.

**Table 1:** Parameters definition.

Parameter	Interpretation
$\beta _i$	The average exposure rate that the susceptible users can contact the information *i*
$\beta _j$	The average exposure rate that the susceptible users can contact the information *j*
$p_i$	The probability that the exposed user will forward the information *i*
$p_j$	The probability that the exposed user will forward the information *j*
$m_{ij}$	The strong attractive index that a forwarding user of state $F_i$ becomes a forwarding user of state $F_{ij}$
$m_{ji}$	The strong attractive index that a forwarding user of state $F_j$ becomes a forwarding user of state $F_{ji}$
$m_{\bar{i}j}$	The continuous attractive index that an immune user of state $I_i$ becomes a forwarding user of state $F_{\bar{i}j}$
$m_{\bar{j}i}$	The continuous attractive index that an immune user of state $I_j$ becomes a forwarding user of state $F_{\bar{j}i}$
$\alpha _i$	The average rate at which a user in the forwarding state of information *i* becomes inactive to forwarding, where 1/$\alpha _i$ is the average duration a forwarding user remains active in forwarding information *i*
$\alpha _j$	The average rate at which a user in the forwarding state of information *j* becomes inactive to forwarding, where 1/$\alpha _j$ is the average duration a forwarding user remains active in forwarding information *j*
$\alpha _{ij}$	The average rate at which a user in the state $F_{\bar{i}j}$ move to the state *I* that immune to both information, usually 1/$\alpha _{ij}$ is the average duration from state $F_{\bar{i}j}$ to immune state *I*
$\alpha _{ji}$	The average rate at which a user in the state $F_{\bar{j}i}$ move to the state *I* that immune to both information usually 1/$\alpha _{ji}$ is the duration from state $F_{\bar{j}i}$ to immune state *I*

## Model formulation

We assume a principle of basic SFI-model^29^ for cross-transmission of two related information co-propagation shown in Fig. 3. Our cross-transmission susceptible-forwarding-immune (CT-SFI) model takes the form in (1).

1 $$\begin{aligned} \left\{ \begin{array}{l} dS(t)/dt =-\beta _i S(t)( F_i( t)+ F_{ij}( t)+ F_{ji}( t)+ F_{\bar{j}i}( t)+ F_{i\bar{j}}( t))-\beta _j S(t)( F_j( t)+ F_{ji}( t)+ F_{ij}( t)+ F_{\bar{i}j}( t)+ F_{j\bar{i}}( t)),\\ dF_i(t)/dt=p_i\beta _i S(t)( F_i( t)+ F_{ij}( t)+ F_{ji}( t)+ F_{\bar{j}i}( t)+ F_{i\bar{j}}( t))-\beta _j F_i(t)( F_j( t)+ F_{ji}( t)+ F_{ij}( t)+ F_{\bar{i}j}( t)+ F_{j\bar{i}}( t))-\alpha _i F_i(t),\\ dF_j(t)/dt=p_j\beta _j S(t)( F_j( t)+ F_{ji}( t)+ F_{ij}( t)+ F_{\bar{i}j}( t)+ F_{j\bar{i}}( t))-\beta _i F_j(t)( F_i( t)+ F_{ij}( t)+ F_{ji}( t)+ F_{\bar{j}i}( t)+ F_{i\bar{j}}( t))-\alpha _j F_j(t),\\ dI_i(t)/dt=(1-p_i)\beta _i S(t)( F_i( t)+ F_{ij}( t)+ F_{ji}( t)+ F_{\bar{j}i}( t)+ F_{i\bar{j}}( t))+\alpha _i F_i(t)-\beta _j I_i(t)( F_j( t)+ F_{ji}( t)+ F_{ij}( t)+ F_{\bar{i}j}( t)+ F_{j\bar{i}}( t)),\\ dI_j(t)/dt=(1-p_j)\beta _j S(t)( F_j( t)+ F_{ji}( t)+ F_{ij}( t)+ F_{\bar{i}j}( t)+ F_{j\bar{i}}( t))+\alpha _j F_j(t)-\beta _i I_j(t)( F_i( t)+ F_{ij}( t)+ F_{ji}( t)+ F_{\bar{j}i}( t)+ F_{i\bar{j}}( t)),\\ dF_{ij}(t)/dt=m_{ij}p_j\beta _j F_i(t)( F_j( t)+ F_{ji}( t)+ F_{ij}( t)+ F_{\bar{i}j}( t)+ F_{j\bar{i}}( t))-\alpha _i F_{ij}(t),\\ dF_{ji}(t)/dt=m_{ji}p_i\beta _i F_j(t)( F_i( t)+ F_{ij}( t)+ F_{ji}( t)+ F_{\bar{j}i}( t)+ F_{i\bar{j}}( t))-\alpha _j F_{ji}(t),\\ dF_{\bar{i}j}(t)/dt=m_{\bar{i}j} p_j\beta _j I_i(t)( F_j( t)+ F_{ji}( t)+ F_{ij}( t)+ F_{\bar{i}j}( t)+ F_{j\bar{i}}( t))+\alpha _i F_{ij}(t)-\alpha _{ij} F_{\bar{i}j}(t),\\ dF_{\bar{j}i}(t)/dt=m_{\bar{j}i} p_i\beta _i I_j(t)( F_i( t)+ F_{ij}( t)+ F_{ji}( t)+ F_{\bar{j}i}( t)+ F_{i\bar{j}}( t))+\alpha _j F_{ji}(t)-\alpha _{ji} F_{\bar{j}i}(t),\\ dF_{i\bar{j}}(t)/dt=(1-m_{ij}p_j)\beta _j F_i(t)( F_j( t)+ F_{ji}( t)+ F_{ij}( t)+ F_{\bar{i}j}( t)+ F_{j\bar{i}}( t))-\alpha _i F_{i\bar{j}}(t),\\ dF_{j\bar{i}}(t)/dt=(1-m_{ji}p_i)\beta _i F_j(t)( F_i( t)+ F_{ij}( t)+ F_{ji}( t)+ F_{\bar{j}i}( t)+ F_{i\bar{j}}( t))-\alpha _j F_{j\bar{i}}(t),\\  \begin{aligned}   dI(t)/dt &=   (1 - m_{{\bar{i}j}} p_{j} )\beta _{j} I_{i} (t)(F_{j} (t) + F_{{ji}} (t) + F_{{ij}} (t) + F_{{\bar{i}j}} (t) + F_{{j\bar{i}}} (t)) + (1 - m_{{\bar{j}i}} p_{i} )\beta _{i} I_{j} (t)(F_{i} (t) + F_{{ij}} (t) \\     &\quad+   F_{{ji}} (t) + F_{{\bar{j}i}} (t) + F_{{i\bar{j}}} (t)) + \alpha _{{ij}} F_{{\bar{i}j}} (t) + \alpha _{{ji}} F_{{\bar{j}i}} (t) + \alpha _{j} F_{{j\bar{i}}} (t) + \alpha _{i} F_{{i\bar{j}}} (t). \\  \end{aligned}  . \end{array} \right. \end{aligned}$$

Here, *i* and *j* (*i*=1 or 2, *j*=1 or 2, and $i\not =j$) denotes two different information. In the model, the mass action term can also be interpreted as following four types of transmission process:

### Transmission dynamics of one single information

**Figure 4:**
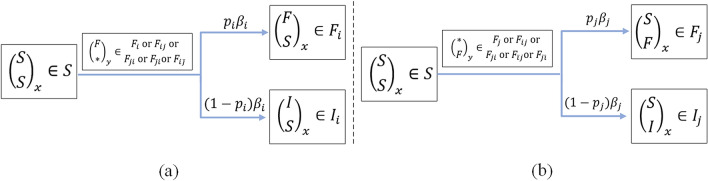
Transmission dynamics of one single information: (**a**) information *i*; (**b**) information *j*.

Figure 4a shows the transmission that a user (user *x*) from a susceptible state $$\left( \begin{array}{c} S\\ S\\ \end{array} \right) _x \in S $$ to a single forwarding state $$\left( \begin{array}{c} F\\ S\\ \end{array} \right) _x \in F_i $$ or a direct immune state $$\left( \begin{array}{c} I\\ S\\ \end{array} \right) _x \in I_i $$ with contacting an active forwarding user (user *y*) with state $$\left( \begin{array}{c} F\\ *\\ \end{array} \right) _y \in F_i $$ or $F_{ij}$ or $F_{ji}$ or $F_{\bar{j}i}$ or $F_{i\bar{j}}$. Considering the whole population, shown in Fig. 3, an active forwarding user of information *i* will contact an average number of $\beta _i N$ users per unit time, among which $p_i \beta _i N$ will choose to forward the information *i* and $(1-p_i) \beta _i N$ will not. Since the probability of a contacted user who is a susceptible user is *S*(*t*)/*N*, there are $F_i( t)+ F_{ij}( t)+ F_{ji}( t)+ F_{\bar{j}i}( t)+ F_{i\bar{j}}( t)$ active forwarding users of information *i* in total at time *t*, then the number of new forwarding and immune users of information *i* are $p_i\beta _i N (S(t)/N)( F_i( t)+ F_{ij}( t)+ F_{ji}( t)+ F_{\bar{j}i}( t)+ F_{i\bar{j}}( t))$ = $p_i\beta _i S(t)( F_i( t)+ F_{ij}( t)+ F_{ji}( t)+ F_{\bar{j}i}( t)+ F_{i\bar{j}}( t))$ and $(1-p_i)\beta _i S(t)( F_i( t)+ F_{ij}( t)+ F_{ji}( t)+ F_{\bar{j}i}( t)+ F_{i\bar{j}}( t))$, respectively, where, $F_i(t)$, $F_{ij}(t)$, $F_{ji}(t)$, $F_{\bar{j}i}(t)$ and $F_{i\bar{j}}(t)$ are five states of active forwarding users for information *i*. Correspondingly, Fig. 4b has the similar transmission process of information *j*.

### Cross-transmission dynamics of two related information in an active forwarding period

**Figure 5:**
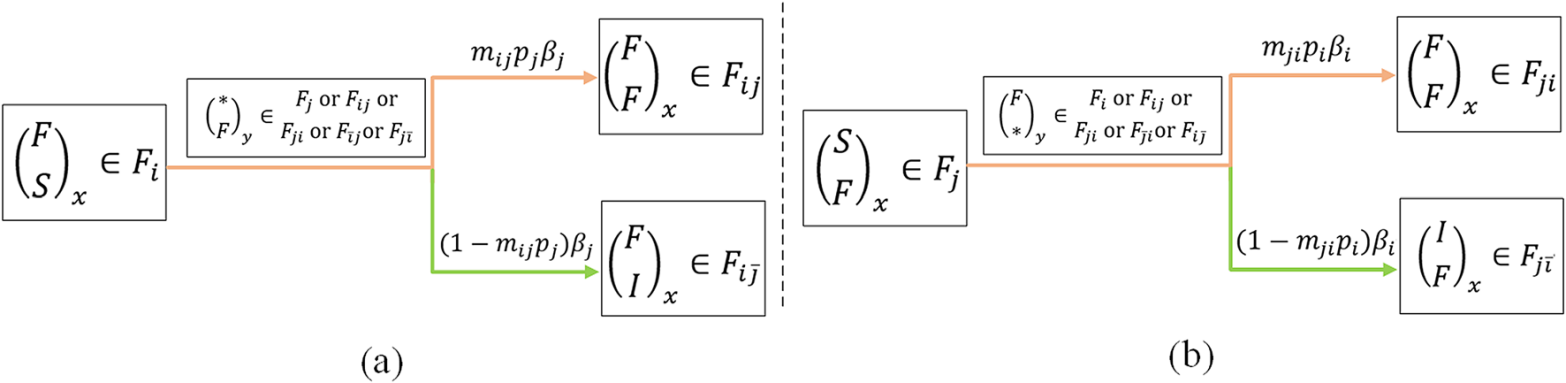
Cross-transmission process of two related information in an active forwarding period: (**a**) forward information *i* then forward information *j* successively; (**b**) forward information *j* then forward information *i* successively.

Figure 5a shows the transmission that a user (user *x*) from a single forwarding state $$\left( \begin{array}{c} F\\ S\\ \end{array} \right) _x \in F_i $$ to a cross-forwarding state $$\left( \begin{array}{c} F\\ F\\ \end{array} \right) _x \in F_{ij} $$ or a partial direct immune state $$\left( \begin{array}{c} F\\ I\\ \end{array} \right) _x \in F_{i\bar{j}} $$ with contacting an active forwarding user (user *y*) with state $$\left( \begin{array}{c} *\\ F\\ \end{array} \right) _y \in F_j $$ or $F_{ij}$ or $F_{ji}$ or $F_{\bar{i}j}$ or $F_{j\bar{i}}$. This is an important distinction between our CT-SFI model and a standard SFI model. Here we analyze and model the cross-transmission of two related information in an active forwarding period. Considering the whole population shown in Fig. 3, in an active forwarding period, the users forward information *i* may be especially attracted by information *j* and have more probability to forward that, we use $m_{ij}$ to measure this strong attraction. Per unit time, an active forwarding user of information *j* will contact an average number of $\beta _j F_i (t)$ users who are in an active forwarding period of information *i*, similar as above, among which $m_{ij} p_j \beta _j F_i (t)$ will choose to forward the information *j* and $(1-m_{ij} p_j)\beta _j F_i (t)$ will not. There are $F_j( t)+ F_{ji}( t)+ F_{ij}( t)+ F_{\bar{i}j}( t)+ F_{j\bar{i}}( t)$ active forwarding users of information *j* in total at time *t*, thus, the number of new forwarding and immune users of information *j* in this process are $m_{ij}p_j\beta _j F_i(t)( F_j( t)+ F_{ji}( t)+ F_{ij}( t)+ F_{\bar{i}j}( t)+ F_{j\bar{i}}( t))$ and $(1-m_{ij}p_j)\beta _j F_i(t)( F_j( t)+ F_{ji}( t)+ F_{ij}( t)+ F_{\bar{i}j}( t)+ F_{j\bar{i}}( t))$, respectively, where, $F_j(t)$, $F_{ji}(t)$, $F_{ij}(t)$, $F_{\bar{i}j}(t)$ and $F_{j\bar{i}}(t)$ are five states of active forwarding users for information *j*. Correspondingly, Fig. 5b shows the similar process of cross-transmission that users firstly forward information *j* and then forward information *i* in an active forwarding period successively.

### Cross-transmission dynamics of two related information within an insensitive period

**Figure 6:**
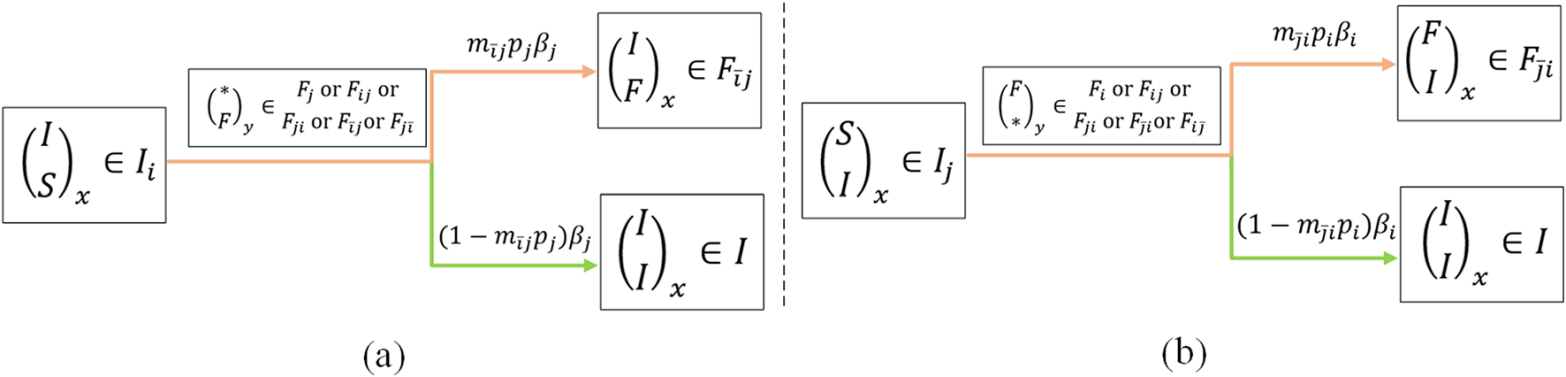
Cross-transmission process of two related information within an insensitive period: (**a**) contact information *i* then forward information *j*; (**b**) contact information *j* then forward information *i*.

Another important co-propagation we considered is the cross-transmission within an insensitive period. Figure 6a shows the transmission that a user (user *x*) from an immune state of information *i* with $$\left( \begin{array}{c} I\\ S\\ \end{array} \right) _x \in I_{i} $$ to a forwarding state of information *j* with $$\left( \begin{array}{c} I\\ F\\ \end{array} \right) _x \in F_{\bar{i}j} $$ or a direct immune state $$\left( \begin{array}{c} I\\ I\\ \end{array} \right) _x \in I $$ with contacting an active forwarding user (user *y*) with state $$\left( \begin{array}{c} *\\ F\\ \end{array} \right) _y \in F_j$$ or $$F_{ij}$$ or $$ F_{ji}$$ or $$F_{\bar{i}j}$$ or $$F_{j\bar{i}}$$. Different from the above cross-transmission in an active forwarding period, the users immune to information *i* may still have a continuous attraction to the other related information *j* because they have contacted the information *i* before, including having already forwarded or gotten direct immunity after exposure subjectively. We use $$m_{\bar{i}j}$$ to measure this continuous attraction. Per unit time, an active forwarding user of information *j*, shown in Fig. 3, will contact an average number of $$\beta _j I_i (t)$$ users who have already contacted before and immune to information *i* now, among which, $$m_{\bar{i}j} p_j \beta _j I_i (t)$$ will choose to forward the information *j* and $$(1-m_{\bar{i}j} p_j) \beta _j I_i (t)$$ will not. In this process, the number of new forwarding and immune user of information *j* are $$m_{\bar{i}j} p_j \beta _j I_i (t)( F_j( t)+ F_{ji}( t)+ F_{ij}( t)+ F_{\bar{i}j}( t)+ F_{j\bar{i}}( t))$$ and $$ (1-m_{\bar{i}j} p_j) \beta _j I_i (t)( F_j( t)+ F_{ji}( t)+ F_{ij}( t)+ F_{\bar{i}j}( t)+ F_{j\bar{i}}( t))$$, respectively. Correspondingly, Fig. 6b shows the similar cross-transmission process that users firstly immune to information *j* within an insensitive period and then forward information *i*.

### Losing an active forwarding state with a period of time

**Figure 7:**
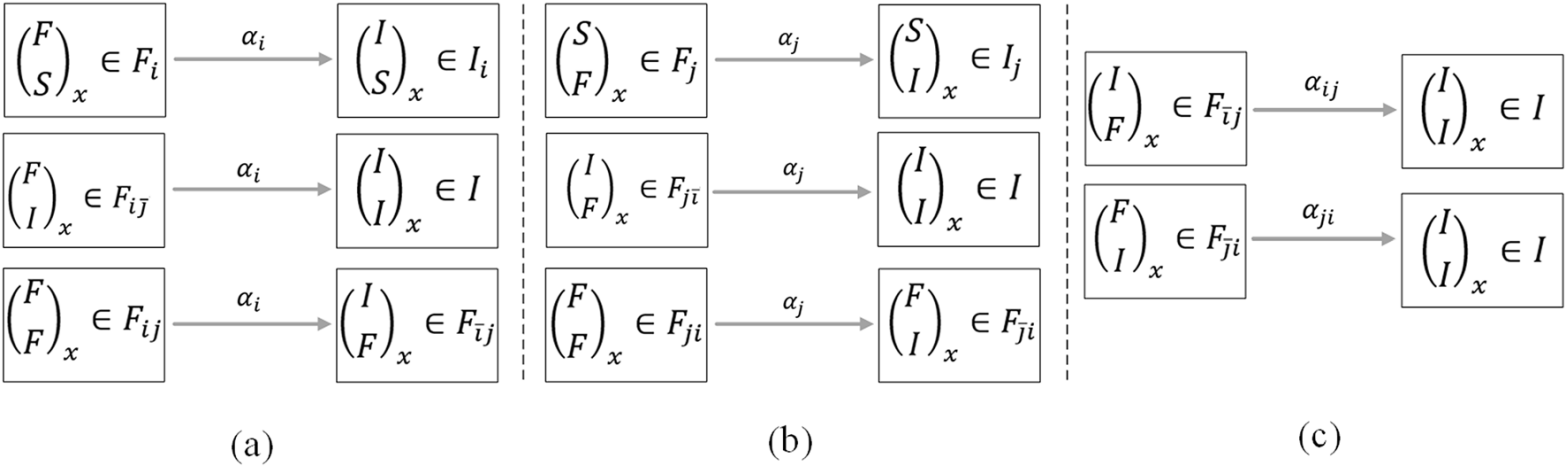
Losing active forwarding state with a period of time: (**a**) information *i*; (**b**) information *j*; (**c**) complex case of information *i* and *j*.

In a dynamic process of information propagation, parameter $$\alpha $$ is the average rate at which a user in the forwarding state becomes inactive to forward. Parameter $$\alpha $$ is usually user-specific because users will not have the patience to find the information out of a certain period so that the information will lose the active ability to be forwarded. And, $$1/\alpha $$ is the average duration a forwarded action remains active in forwarding. In our CT-SFI model, we have eight processes of losing active transmission as shown in Fig. 7. Here, $$\alpha _i$$ denotes the average rate at which a user in the forwarding state of information *i* becomes inactive to forwarding, which reflects the process from $$\left( \begin{array}{c} F\\ S\\ \end{array} \right) _x \in F_{i} $$ to $$\left( \begin{array}{c} I\\ S\\ \end{array} \right) _x \in I_{i} $$, from $$\left( \begin{array}{c} F\\ I\\ \end{array} \right) _x \in F_{i\bar{j}} $$ to $$\left( \begin{array}{c} I\\ I\\ \end{array} \right) _x \in I $$ , and from $$\left( \begin{array}{c} F\\ F\\ \end{array} \right) _x \in F_{ij} $$ to $$\left( \begin{array}{c} I\\ F\\ \end{array} \right) _x \in F_{\bar{i}j} $$ , as shown in Fig. 7a. Correspondingly, $$\alpha _j$$ for the losing active process of information *j* as shown in Fig. 7b. Especially, $$\alpha _{ij}$$ denotes the average rate at which a user in the state $$\left( \begin{array}{c} I\\ F\\ \end{array} \right) _x \in F_{\bar{i}j} $$ moves to the state $$\left( \begin{array}{c} I\\ I\\ \end{array} \right) _x \in I $$ that immune to both information, which reflects the immune process of information *j*. It is different from $$\alpha _i$$ because a part of $$F_{\bar{i}j} (t)$$ comes from $$ F_{ij} (t)$$ and they have a faster immune rate for information *j*. A similar process for $$\alpha _{ji}$$ shown in Fig. 7c.

**Figure 8:**
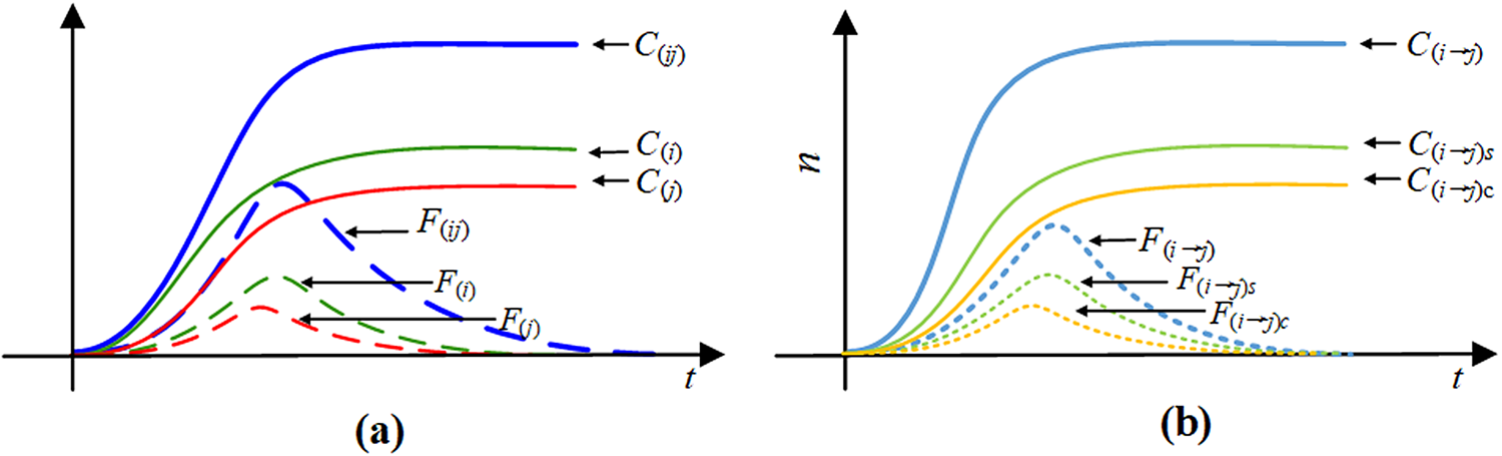
The temporal variation of the cumulative forwarding population and the active forwarding population: (**a**) the summative number of forwarding users; (**b**) the number of cross-transmission users.

Considering the whole dissemination effect of the two related information, we pay more attention to the summative number of forwarding population shown in Fig. 8a. Fortunately, this cumulative number $C_{(ij)} (t)$ (the blue line) consists of two important pieces of information which we can obtain directly from the propagation process, represented by the cumulative forwarding users $C_{(i)} (t)$ for information *i* (the green line) and the cumulative forwarding users $C_{(j)} (t)$ for information *j* (the red line). We have

2 $$\begin{aligned} \begin{array}{c} C_{(ij)} (t) = C_{(i)} (t)+C_{(j)} (t), \end{array} \end{aligned}$$

in which

3 $$\begin{aligned} \left\{ \begin{array}{l} C_{(i)}(t)=\int p_i\beta _i S(t)( F_i( t)+ F_{ij}( t) + F_{ji}( t)+ F_{\bar{j}i}( t)+ F_{i\bar{j}}( t))\,dt\\ \quad+\int m_{ij}p_j\beta _j F_i(t)( F_j( t)+ F_{ji}( t) + F_{ij}( t)+ F_{\bar{i}j}( t)+ F_{j\bar{i}}( t))\,dt\\ \quad+\int m_{ji}p_i\beta _i F_j(t)( F_i( t)+ F_{ij}( t) + F_{ji}( t)+ F_{\bar{j}i}( t)+ F_{i\bar{j}}( t))\,dt\\ \quad+ \int m_{\bar{j}i}p_i\beta _i I_j(t)( F_i( t)+ F_{ij}( t) + F_{ji}( t)+ F_{\bar{j}i}( t)+ F_{i\bar{j}}( t))\,dt\\ \quad+\int (1-m_{ij}p_j)\beta _j F_i(t)( F_j( t)+ F_{ji}( t)+ F_{ij}( t)+ F_{\bar{i}j}( t)+ F_{j\bar{i}}( t))\,dt \end{array} \right. \end{aligned}$$

and

4 $$\begin{aligned} \left\{ \begin{array}{l} C_{(i)}(t)=\int p_j\beta _j S(t)( F_j( t)+ F_{ji}( t) + F_{ij}( t)+ F_{\bar{i}j}( t)+ F_{j\bar{i}}( t))\,dt\\ \quad+\int m_{ji}p_i\beta _i F_j(t)( F_i( t)+ F_{ij}( t) + F_{ji}( t)+ F_{\bar{j}i}( t)+ F_{i\bar{j}}( t))\,dt\\ \quad+\int m_{ij}p_j\beta _j F_i(t)( F_j( t)+ F_{ji}( t) + F_{ij}( t)+ F_{\bar{i}j}( t)+ F_{j\bar{i}}( t))\,dt\\ \quad+\int m_{\bar{i}j} p_j\beta _j I_i(t)( F_j( t)+ F_{ji}( t) + F_{ij}( t)+ F_{\bar{i}j}( t)+ F_{j\bar{i}}( t))\,dt\\ \quad+\int (1-m_{ji}p_i)\beta _i F_j(t)( F_i( t)+ F_{ij}( t)+ F_{ji}( t)+ F_{\bar{j}i}( t)+ F_{i\bar{j}}( t))\,dt. \end{array} \right. \end{aligned}$$

Here, the number of active forwarding users (the blue dotted line) at each time is

5 $$\begin{aligned} \begin{array}{cc} F_{(ij)} (t)=F_{(i)} (t)\cup F_{(j)} (t)=F_i (t)+F_j (t)+F_{ij} (t)+F_{ji} (t)+F_{\bar{j}i} (t)+F_{i\bar{j}} (t)+F_{\bar{i}j} (t)+F_{j\bar{i}} (t), \end{array} \end{aligned}$$

in which

6 $$\begin{aligned} \begin{array}{cc} F_{(i)} (t)=F_i (t)+F_{ij} (t)+F_{ji} (t)+F_{\bar{j}i} (t)+F_{i\bar{j}} (t) \end{array} \end{aligned}$$

and

7 $$\begin{aligned} \begin{array}{cc} F_{(j)} (t)=F_j (t)+F_{ji} (t)++F_{ij} (t)+F_{\bar{i}j} (t)+F_{j\bar{i}} (t) \end{array} \end{aligned}$$

are the forwarding users for information *i* (the green dotted line) and information *j* (the red dotted line) at each time respectively.

We also have a great interest in the cross-transmission population which will help us to analyze the influencing factors and formulate strategies for the co-propagation of the public hot event. We want to know how the parameters influence the cross-transmission and how the cross-transmission contributes to the co-propagation. Figure 8b shows the summative cumulative cross-transmission population $C_{(i\rightarrow j)} (t)$ (the light blue line) originating from the attraction of the information *j* after contacting the information *i*, which consists of two parts: one is the cumulative forwarding number $C_{(i\rightarrow j)s}(t)$ (the light green line) composed of cross-transmission users who are influenced by strong attraction within an active forwarding period of information *i*; the other is the cumulative forwarding number $C_{(i\rightarrow j)c}(t)$ (the yellow line) composed of cross-transmission users who are influenced by continuous attraction within an insensitive period of information *i*. Given by

8 $$\begin{aligned} \begin{array}{cc} C_{(i\rightarrow j)}(t)=C_{(i\rightarrow j)s}(t)+C_{(i\rightarrow j)c}(t), \end{array} \end{aligned}$$

in which

9 $$\begin{aligned} \begin{array}{cc} C_{(i\rightarrow j)s}(t)=\int m_{ij}p_j\beta _j F_i(t)( F_j( t)+ F_{ji}( t)+ F_{ij}( t)+ F_{\bar{i}j}( t)+ F_{j\bar{i}}( t))\,dt \end{array} \end{aligned}$$

and

10 $$\begin{aligned} \begin{array}{cc} C_{(i\rightarrow j)c}(t) =\int m_{\bar{i}j} p_j\beta _j I_i(t)( F_j( t)+ F_{ji}( t)+ F_{ij}( t)+ F_{\bar{i}j}( t)+ F_{j\bar{i}}( t))\,dt. \end{array} \end{aligned}$$

Correspondingly, the number of cross-transmission users (the light blue dotted line) originating from the attraction of the information *j* after contacting the information *i* at each time is

11 $$\begin{aligned} \begin{array}{cc} F_{(i\rightarrow j)}(t)=F_{(i\rightarrow j)s}(t)+F_{(i\rightarrow j)c}(t), \end{array} \end{aligned}$$

in which

12 $$\begin{aligned} \begin{array}{cc} F_{(i\rightarrow j)s}(t)=F_{ij} (t) \end{array} \end{aligned}$$

and

13 $$\begin{aligned} \begin{array}{cc} F_{(i\rightarrow j)c}(t)=F_{\bar{i}j} (t) \end{array} \end{aligned}$$

are two components. The instantaneous forwarding number $F_{(i\rightarrow j)s}(t)$ (light green dotted line) is composed of cross-transmission users who are influenced by strong attraction within an active forwarding period of information *i*, and the instantaneous forwarding number $F_{(i\rightarrow j)c}(t)$ (the yellow dotted line) is composed of cross-transmission users who are influenced by continuous attraction within an insensitive period of information *i*.

For the symmetry of our CT-SFI model, the character of the summative cumulative cross-transmission population $C_{(j\rightarrow i)}(t)=C_{(j\rightarrow i)s}(t)+C_{(j\rightarrow i)c}(t)$ and the active forwarding population $F_{(j\rightarrow i)}(t)=F_{(j\rightarrow i)s}(t)+F_{(j\rightarrow i)c}(t)$ originating from the attraction of the information *i* after contacting the information *j* is similar to that originating from the attraction of the information *j* after contacting the information *i*.

## Co-propagation indices

### The public opinion reproduction ratio

In the epidemic model^30^, $\mathfrak {R}_{0}$ is the basic reproduction ratio which denotes the average number of secondary cases infected by a patient during the average infectious period. Similarly, in our CT-SFI model, we use the public opinions reproduction ratios $\mathfrak {R}_{0}$ to evaluate the outbreak of the public hot event. We follow the calculation of basic reproduction number developed in Ref.^24^, and rewrite our model system (1) as the following form

14 $$\begin{aligned} \begin{array}{cc} {\dot{x}}={\mathcal {M}}(x)-{\mathcal {V}}(x), \end{array} \end{aligned}$$

where $$x=(F_i (t),F_j (t),F_{ij} (t),F_{ji} (t),F_{\bar{i}j}(t),F_{\bar{j}i} (t),F_{i\bar{j}} (t),F_{j\bar{i}} )^{T}$$ and

15 $$\begin{aligned}&{\mathcal {M}}(x)= \left\{ \begin{array}{l} p_i\beta _i S(t)( F_i( t)+ F_{ij}( t)+ F_{ji}( t) + F_{\bar{j}i}( t)+ F_{i\bar{j}}( t))\\ p_j\beta _j S(t)( F_j( t)+ F_{ji}( t)+ F_{ij}( t) + F_{\bar{i}j}( t)+ F_{j\bar{i}}( t))\\ m_{ij}p_j\beta _j F_i(t)( F_j( t)+ F_{ji}( t)+ F_{ij}( t) + F_{\bar{i}j}( t)+ F_{j\bar{i}}( t))\\ m_{ji}p_i\beta _i F_j(t)( F_i( t)+ F_{ij}( t)+ F_{ji}( t) + F_{\bar{j}i}( t)+ F_{i\bar{j}}( t))\\ m_{\bar{i}j}p_j\beta _j I_i(t)( F_j( t)+ F_{ji}( t)+ F_{ij}( t) + F_{\bar{i}j}( t)+ F_{j\bar{i}}( t))\\ m_{\bar{j}i}p_i\beta _i I_j(t)( F_i( t)+ F_{ij}( t)+ F_{ji}( t) + F_{\bar{j}i}( t)+ F_{i\bar{j}}( t))\\ (1-m_{ij}p_j)\beta _j F_i(t)( F_j( t)+ F_{ji}( t)+ F_{ij}( t) + F_{\bar{i}j}( t)+ F_{j\bar{i}}( t))\\ (1-m_{ji}p_i)\beta _i F_j(t)( F_i( t)+ F_{ij}( t)+ F_{ji}( t)+ F_{\bar{j}i}( t)+ F_{i\bar{j}}( t)) \end{array} \right. \end{aligned}$$

16 $$\begin{aligned}&{\mathcal {V}}(x)= \left\{ \begin{array}{l} \beta _j F_i(t)( F_j( t)+ F_{ji}( t)+ F_{ij}( t) + F_{\bar{i}j}( t)+ F_{j\bar{i}}( t))+\alpha _i F_{i}( t)\\ \beta _i F_j(t)( F_i( t)+ F_{ij}( t)+ F_{ji}( t) + F_{\bar{j}i}( t)+ F_{i\bar{j}}( t))+\alpha _j F_{j}( t)\\ \alpha _i F_{ij}( t)\\ \alpha _j F_{ji}( t)\\ -\alpha _i F_{ij}( t)+\alpha _{ij} F_{\bar{i}j}( t)\\ -\alpha _j F_{ji}( t)+\alpha _{ji} F_{\bar{j}i}( t)\\ \alpha _i F_{i\bar{j}}( t)\\ \alpha _j F_{j\bar{i}}( t) \end{array} \right. \end{aligned}$$

It is apparent that our model (1) always has a no information propagation equilibrium $F_0=(S_0,0,0,0,0,0,0,0,0,0,0,0).$ Calculate the derivatives *M* and *V* at no information propagation equilibrium, we have

17 $$\begin{aligned} M= \left[ \begin{array}{cccccccc} p_i\beta _i S_0 &{} 0 &{} p_i\beta _i S_0 &{} p_i\beta _i S_0 &{} 0 &{} p_i\beta _i S_0 &{} p_i\beta _i S_0 &{} 0 \\ 0 &{} p_j\beta _j S_0 &{} p_j\beta _j S_0 &{} p_j\beta _j S_0 &{} p_j\beta _j S_0 &{} 0 &{} 0 &{} p_j\beta _j S_0 \\ 0 &{} 0 &{} 0 &{} 0 &{} 0 &{} 0 &{} 0 &{} 0 \\ 0 &{} 0 &{} 0 &{} 0 &{} 0 &{} 0 &{} 0 &{} 0 \\ 0 &{} 0 &{} 0 &{} 0 &{} 0 &{} 0 &{} 0 &{} 0 \\ 0 &{} 0 &{} 0 &{} 0 &{} 0 &{} 0 &{} 0 &{} 0 \\ 0 &{} 0 &{} 0 &{} 0 &{} 0 &{} 0 &{} 0 &{} 0 \\ 0 &{} 0 &{} 0 &{} 0 &{} 0 &{} 0 &{} 0 &{} 0 \\ \end{array} \right] \end{aligned}$$

and

18 $$\begin{aligned} V= \left[ \begin{array}{cccccccc} \alpha _i &{} 0 &{} 0 &{} 0 &{} 0 &{} 0 &{} 0 &{} 0 \\ 0 &{}\alpha _j &{} 0 &{} 0 &{} 0 &{} 0 &{} 0 &{} 0 \\ 0 &{} 0 &{}\alpha _i&{}0 &{} 0 &{} 0 &{} 0 &{} 0 \\ 0 &{} 0 &{} 0 &{}\alpha _j &{} 0 &{} 0 &{} 0 &{} 0 \\ 0 &{} 0 &{} -\alpha _i &{} 0&{}\alpha _{ij} &{} 0 &{} 0 &{} 0 \\ 0 &{} 0 &{} 0 &{} -\alpha _j &{} 0&{}\alpha _{ji} &{} 0 &{} 0 \\ 0 &{} 0 &{} 0 &{} 0 &{} 0 &{} 0 &{} \alpha _i &{} 0 \\ 0 &{} 0 &{} 0 &{} 0 &{} 0 &{} 0 &{} 0 &{}\alpha _j \\ \end{array} \right] . \end{aligned}$$

Thereby

19 $$\begin{aligned} V^{-1}= \left[ \begin{array}{cccccccc} \frac{1}{\alpha _i} &{} 0 &{} 0 &{} 0 &{} 0 &{} 0 &{} 0 &{} 0 \\ 0 &{}\frac{1}{\alpha _j} &{} 0 &{} 0 &{} 0 &{} 0 &{} 0 &{} 0 \\ 0 &{} 0 &{}\frac{1}{\alpha _i}&{}0 &{} 0 &{} 0 &{} 0 &{} 0 \\ 0 &{} 0 &{} 0 &{}\frac{1}{\alpha _j} &{} 0 &{} 0 &{} 0 &{} 0 \\ 0 &{} 0 &{} \frac{1}{\alpha _{ij}} &{} 0&{}\frac{1}{\alpha _{ij}} &{} 0 &{} 0 &{} 0 \\ 0 &{} 0 &{} 0 &{} \frac{1}{\alpha _{ji}} &{} 0&{}\frac{1}{\alpha _{ji}} &{} 0 &{} 0 \\ 0 &{} 0 &{} 0 &{} 0 &{} 0 &{} 0 &{} \frac{1}{\alpha _i} &{} 0 \\ 0 &{} 0 &{} 0 &{} 0 &{} 0 &{} 0 &{} 0 &{}\frac{1}{\alpha _j} \\ \end{array} \right] , \end{aligned}$$

and we can get

20 $$\begin{aligned} MV^{-1}= \left[ \begin{array}{cccccccc} \frac{p_i\beta _i S_0}{\alpha _i} &{} 0 &{} \frac{p_i\beta _i S_0}{\alpha _i} &{} \frac{p_i\beta _i S_0}{\alpha _j}+\frac{p_i\beta _i S_0}{\alpha _{ji}} &{} 0 &{} \frac{p_i\beta _i S_0}{\alpha _{ji}} &{} \frac{p_i\beta _i S_0}{\alpha _i} &{} 0 \\ 0 &{} \frac{p_j\beta _j S_0}{\alpha _j} &{} \frac{p_j\beta _j S_0}{\alpha _i} +\frac{p_j\beta _j S_0}{\alpha _{ij}} &{} \frac{p_j\beta _j S_0}{\alpha _j} &{} \frac{p_j\beta _j S_0}{\alpha _{ij}} &{} 0 &{} 0 &{} \frac{p_j\beta _j S_0}{\alpha _j} \\ 0 &{} 0 &{} 0 &{} 0 &{} 0 &{} 0 &{} 0 &{} 0 \\ 0 &{} 0 &{} 0 &{} 0 &{} 0 &{} 0 &{} 0 &{} 0 \\ 0 &{} 0 &{} 0 &{} 0 &{} 0 &{} 0 &{} 0 &{} 0 \\ 0 &{} 0 &{} 0 &{} 0 &{} 0 &{} 0 &{} 0 &{} 0 \\ 0 &{} 0 &{} 0 &{} 0 &{} 0 &{} 0 &{} 0 &{} 0 \\ 0 &{} 0 &{} 0 &{} 0 &{} 0 &{} 0 &{} 0 &{} 0 \\ \end{array} \right] . \end{aligned}$$

The roots of the characteristic equation can deduce the eigenvalues of the matrix $MV^{-1}$:

21 $$\begin{aligned} \begin{array}{cc} |\lambda E-MV^{-1}|=\lambda ^{6}(\lambda -\frac{p_i\beta _i S_0}{\alpha _i})(\lambda -\frac{p_j\beta _j S_0}{\alpha _j})=0. \end{array} \end{aligned}$$

The reproduction ratio $\mathfrak {R}_0$, defined as the outbreak potential of the public hot event, is the spectral radium of $\rho (MV^{-1}$). Therefore, we have

22 $$\begin{aligned} \begin{array}{cc} \mathfrak {R}_0=max \left\{ {\frac{p_i\beta _i S_0}{\alpha _i},\frac{p_j\beta _j S_0}{\alpha _j}} \right\} . \end{array} \end{aligned}$$

And when $\mathfrak {R}_0 < 1$, the number of forwarding users decreases repidly and hence the propagation would never break out. However, when $\mathfrak {R}_0 > 1$ the forwarding population grows exponentially initially.

### The co-propagation indices

**Figure 9:**
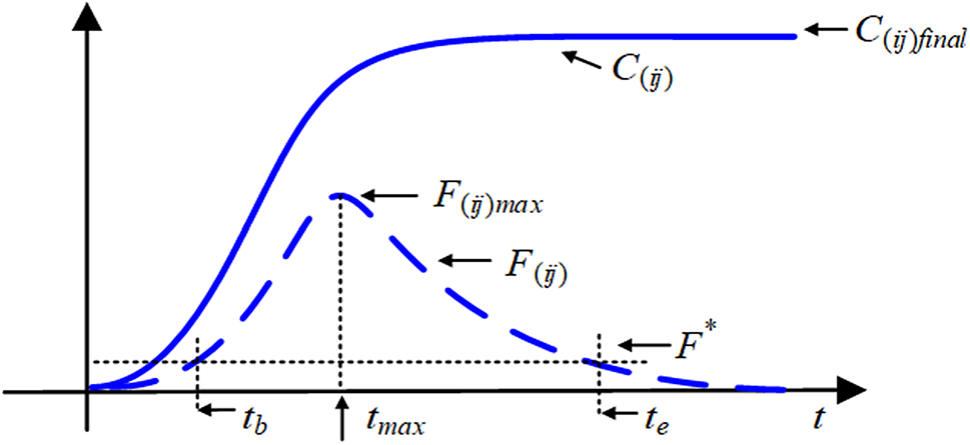
Some summative indices of the public hot event.

Considering the process of co-propagation, some indices of interest will be used in the subsequent studies, as shown in Fig. 9.

- The outbreak peak $F_{{(ij)}max}$: the maximum of curve $F_{(ij)}$, which reflects the peak of the public hot event.
- The final size $C_{{(ij)}final}$: the stable state of curve $C_{(ij)}$, which reflects the final scale of the public hot event.
- The outbreak time $t_b$, the end time $t_e$ and the duration $t_d$: the definition depends on the threshold $F^*$ set in advance, so that $F(t_b )=F^*=F(t_e)$. Here, $t_b$ denotes the outbreak time of the public hot event, $t_e$ denotes the end time, and $t_d=t_e-t_b$ denotes the duration of this event. These three-time indices will help us judge the stage of co-propagation.
- The outbreak velocity $V_o$ and the declining velocity $V_d$: the definition depends on $V_o=(F_{max}-F^*)/(t_{max}-t_b)$ and $V_d=(F_{max}-F^*)/(t_e-t_{max})$ when $F_{(ij)} (t)=F_{{(ij)}max}$ and $t_{max}$ is definite, which reflects the speed of the outbreak and the decline of the public hot event.

## Data fitting

**Figure 10:**
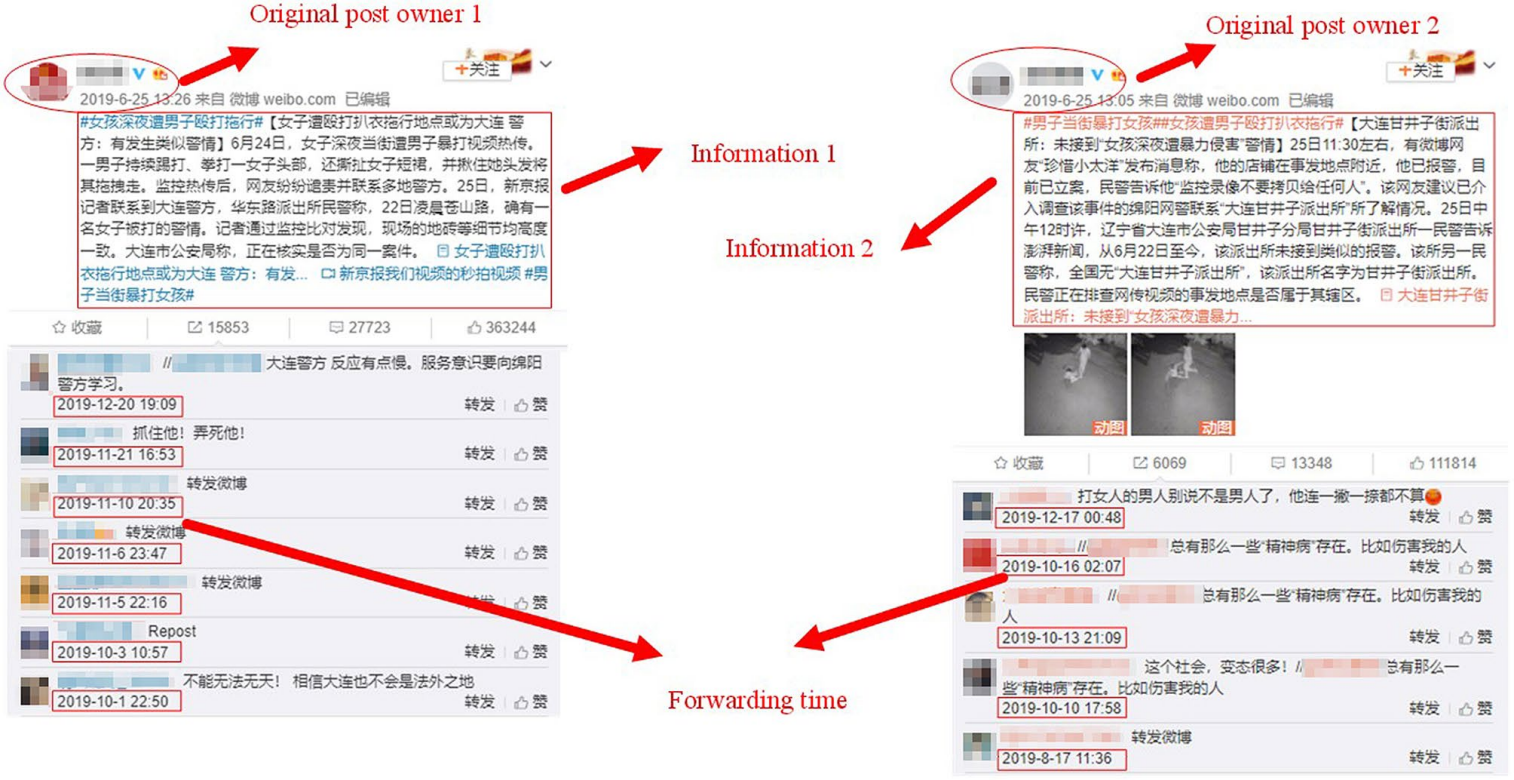
The source of data collection for one public hot event.

In this paper, we use the real data from Chinese Sina-Microblog as shown in Fig. 10 to verify the effectiveness of our CT-SFI model. The data of the precise time of the forwarding record is collected from the Application Program Interface (API). We filter the raw data to avoid the limitation of information stagnation caused by physiological need, and obtain the cumulative forwarding number of the information. The beginning time when the event outbreaks is set to 0 and the sampling frequency is set to 10 min.

Tables 2 and 3 give two typical public hot events driven by two related information. For public hot event 1, # A girl was killed by the driver of a DiDi carpool #, information *i* spread slower at the beginning but had a longer outburst duration than the other information. For public hot event 2 which caused widespread concern and discussion as well, # A man beat up a girl in the street #, information *i* spread in a slower speed at the beginning and was less popular than information *j*.

To use our CT-SFI model to explore some distinctive behaviors, we use the LS method to estimate the model parameters and the initial susceptible population. The parameter vector can be set as $\Theta =(\beta _i,\beta _j,p_i,p_j,\alpha _i,\alpha _j,\alpha _{ij}, \alpha _{ji},m_{ij},m_{ji},m_{\bar{i}j},m_{\bar{j}i} ,S_0).$

The LS error function

23 $$\begin{aligned} { LS}=\sum _{k=0}^T\left| {{C_{(i)}}( t_k,\Theta )}-{ x_{ik}} \right| ^2+\sum _{k=0}^T\left| {{C_{(j)}}( t_k,\Theta )}-{ x_{jk}} \right| ^2 \end{aligned}$$

is used in our calculation, where $C_{(i)}( t_k,\Theta )$ and $C_{(j)}( t_k,\Theta )$ represent the corresponding numerical solution for our model under the given parameter $\theta$; $x_{ik}$ and $x_{jk}$ denote the actual cumulative forwarding number given in Tables 2 and 3; ${ t_k}={ k}$ is the sampling time, ${ k}=0,1,2,\ldots$.

**Table 2:** Two cumulative forwarding number of public hot event 1-“A girl was killed by a taxi (DiDi in China) driver”, Aug.25th, 2018, a 20-year-old girl was raped and killed by a taxi driver in Yueqing, Zhejiang province.

t(10 min)	0	1	2	3	4	5	6	7	8	9
Information 1	138	288	633	1046	1448	1931	2438	2935	3385	3796
Information 2	196	381	508	629	753	819	871	940	1014	1109
t(10 min)	10	11	12	13	14	15	16	17	18	19
Information 1	4194	4595	4782	4895	4972	5039	5113	5157	5214	5252
Information 2	1213	1303	1411	1505	1607	1710	1790	1878	1965	2057
t(10 min)	20	21	22	23	24	25	26	27	28	29
Information 1	5286	5306	5315	5326	5342	5351	5363	5376	5382	5394
Information 2	2250	2463	2653	2848	3021	3205	3371	3564	3749	3909
t(10 min)	30	31	32	33	34	35	36	37	38	39
Information 1	5406	5418	5429	5441	5448	5456	5471	5476	5481	5482
Information 2	4079	4246	4412	4601	4753	4933	5086	5273	5456	5596
t(10 min)	40	41	42	43	44	45	46	47	48	49
Information 1	5483	5484	5490	5494	5499	5500	5512	5515	5520	5528
Information 2	5736	5898	6057	6132	6191	6239	6281	6331	6365	6396
t(10 min)	50	51	52	53	54					
Information 1	5533	5538	5542	5552	5559					
Information 2	6422	6462	6498	6529	6552					

At 11:23 a.m., information 1 about the girl had asked a friend for help before she was killed appeared at a lower exposure, nearly at the same time, information 2 about the murderer had been caught burst at a relatively high exposure, and both information was posted by @*Toutiaonews*.

**Table 3:** Two cumulative forwarding number of public hot event 2-“A man beat up a girl in the street”, Jun.25th, 2019.

t(10 min)	0	1	2	3	4	5	6	7	8	9
Information 1	344	384	434	507	544	563	618	759	840	1007
Information 2	579	1272	2370	3397	4293	4932	5516	6021	6465	6791
t(10 min)	10	11	12	13	14	15	16	17	18	19
Information 1	1159	1289	1414	1819	2184	2440	2592	2684	2782	2889
Information 2	7067	7250	7401	7704	7887	8029	8158	8420	8705	8941
t(10 min)	20	21	22	23	24	25	26	27	28	29
Information 1	2960	3040	3152	3256	3363	3475	3540	3554	3567	3656
Information 2	9164	9386	9590	9855	10139	10405	10582	10623	10673	10842
t(10 min)	30	31	32	33	34	35	36	37	38	39
Information 1	3683	3713	3768	3831	3900	3984	4051	4112	4179	4247
Information 2	10902	10949	11083	11238	11406	11575	11718	11835	11999	12175
t(10 min)	40	41	42	43	44	45	46	47	48	49
Information 1	4303	4372	4423	4489	4549	4589	4636	4680	4722	4766
Information 2	12325	12475	12612	12741	12868	12988	13095	13095	13291	13388
t(10 min)	50	51	52	53	54	55	56	57	58	59
Information 1	4820	4863	4922	4956	5001	5040	5071	5107	5130	5180
Information 2	13486	13573	13670	13747	13844	13943	14028	14107	14183	14257
t(10 min)	60	61	62	63	64	65	66	67	68	69
Information 1	5223	5223	5223	5223	5223	5223	5223	5223	5223	5225
Information 2	14265	14270	14273	14277	14286	14288	14293	14293	14297	14298
t(10 min)	70	71	72	73	74	75	76	77	78	79
Information 1	5225	5226	5227	5228	5230	5254	5267	5270	5270	5271
Information 2	14301	14303	14304	14308	14339	14368	14370	14372	14373	14374
t(10 min)	80	81	82	83	84	85	86	87		
Information 1	5274	5274	5274	5274	5274	5274	5274	5274		
Information 2	14374	14376	14376	14378	14381	14381	14381	14381		

On the streets of Dalian, Wu, the victim, was beaten by a man on her way home at night. At 1:06 p.m., information 1 about where the event occurred posted by @*TheBeijingnews* burst slowly, after a short period, information 2 bursting with a great attention posted by @*Thepaper* was that police did not get a call after the incident.

**Figure 11:**
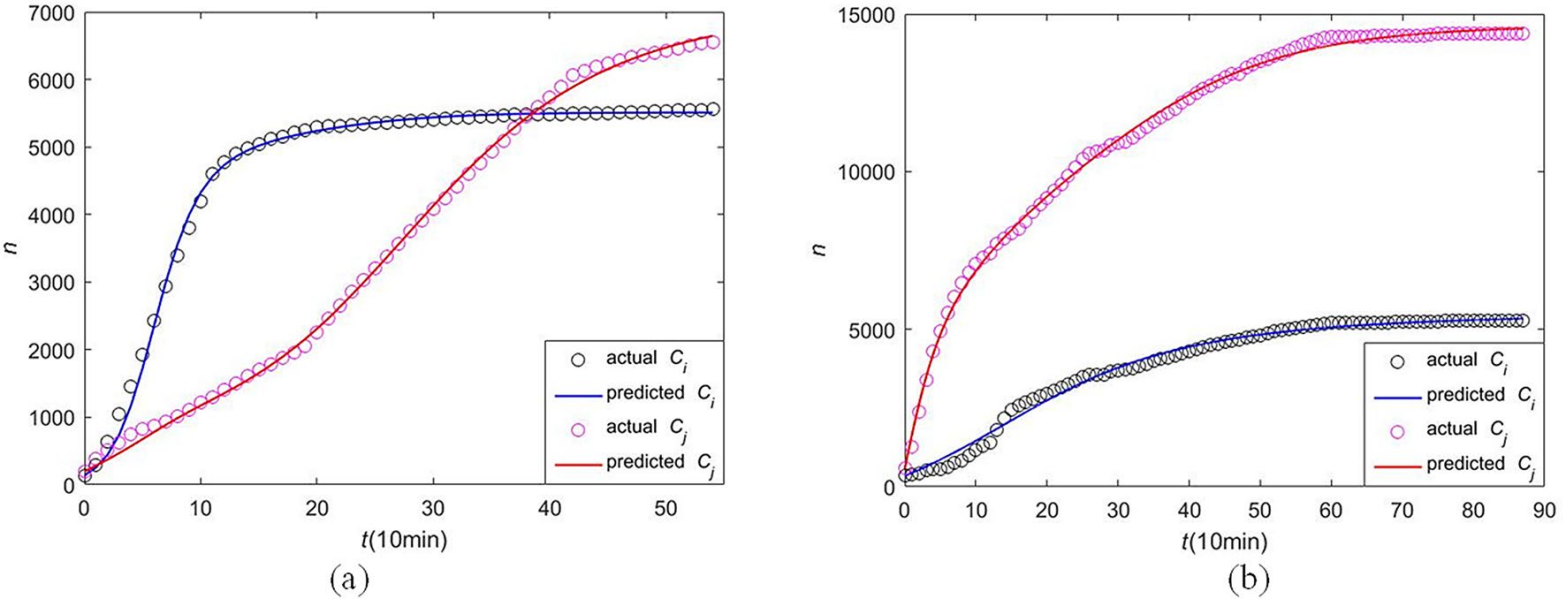
Numerical data fitting results of two public hot events. (**a**) event 1 in Table 2; (**b**) event 2 in Table 3. Where the black dot is the actual cumulative forwarding population and the blue line is the results of data fitting of information *i*; the pink dot is the actual cumulative forwarding population and the red line is the results of data fitting of information *j*.

A good data fitting result shown in Fig.11 reflects that our CT-SFI model has a good effect on the modeling for cross-transmission of co-propagation. For both event 1 “close heats” and event 2 “discriminating heats”, our CT-SFI model can achieve data fitting well, demonstrating the effectiveness of the model, which could apply not only to Chinese Sina Microblog but also to other platforms with similar attributes such as Twitter.

**Figure 12:**
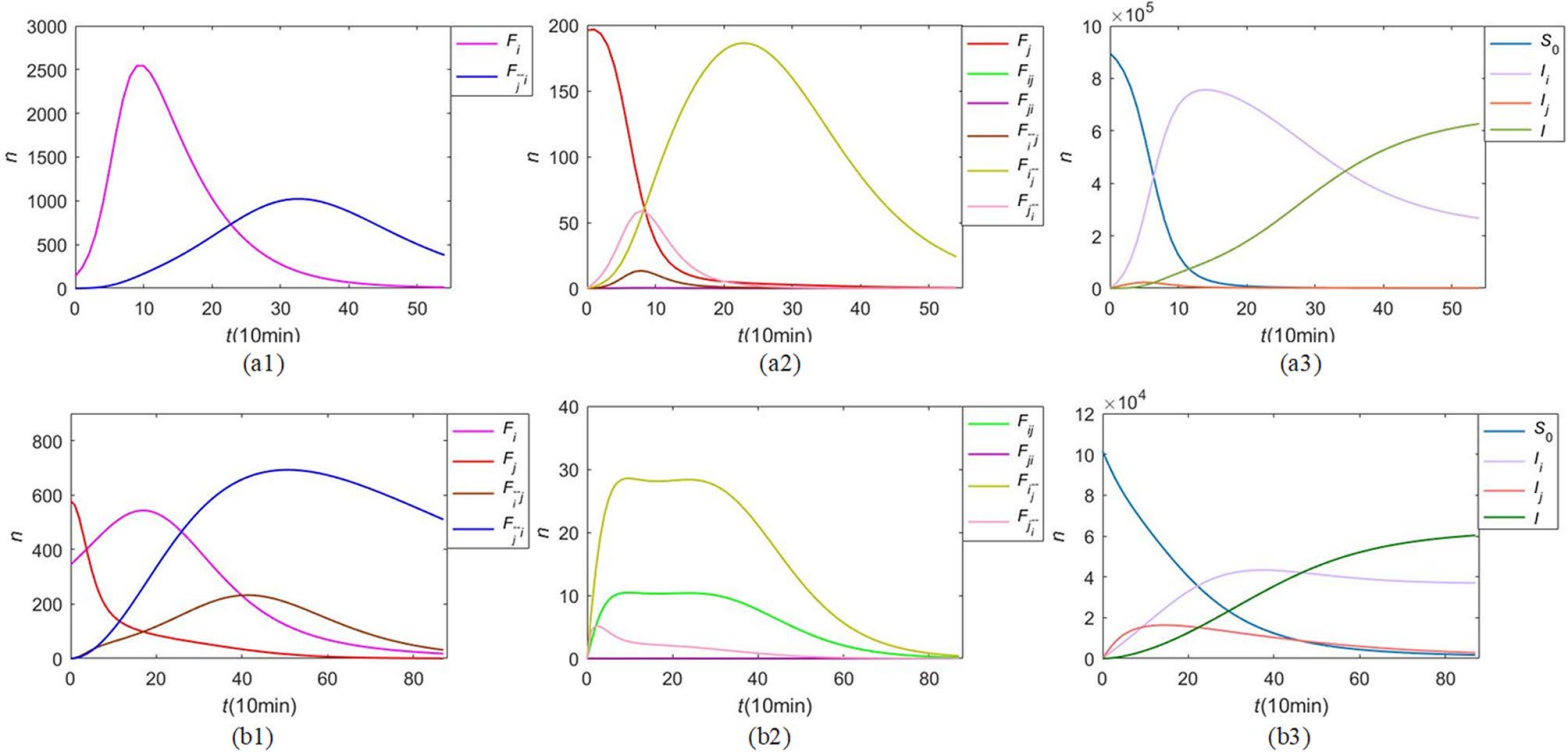
Physical process of CT-SFI model. (**a**) event 1 in Table 2; (**b**) event 2 in Table 3. Where *S*(*t*), $F_{i}(t)$, $F_{j}(t)$, $I_{i}(t)$, $I_{j}(t)$, $F_{ij} (t)$, $F_{ji} (t)$, $F_{\bar{i}j} (t)$, $F_{\bar{j}i} (t)$, $F_{i\bar{j}} (t)$, $F_{j\bar{i}} (t)$, *I*(*t*) are represented by different colored lines, and similar orders of magnitude of the population are put on the same graph.

Figure 12 shows the physical process of each population based on our CT-SFI model during the whole duration, where (a1) (a2) (a3) are the physical processes of event 1 and (b1) (b2) (b3) are the physical processes of event 2. From Fig.12a1–a2 and b1–b2, we can obtain the process of forwarding population that both $F_{i}(t)$ and $F_{\bar{j}i} (t)$ contribute the most to the forwarding number, and $F_{ij} (t)$, $F_{ji} (t)$, $F_{i\bar{j}} (t)$ and $F_{j\bar{i}} (t)$ have a very slight effect on the cross-transmission of two events. In addition, $F_{j}(t)$ and $F_{\bar{i}j} (t)$ are also important to the forwarding number for co-propagation of event 2, but the effect on event 1 is negligible. Fig. 12a3 and b3 shows the process of the susceptible population and immune population, in which $I_{i}$ and $I_{j}$ will have an upward trend in the initial stage, then more and more users will leave to other states after contacting another information, therefore it tends to be bell-shaped. The integrated *I* shows that the curve will rise and tends to *N* when each population is immune to the overall event gradually. The curve *S* of susceptible population is downward and tends to 0. According to the propagation events with different characteristics, different forwarding population has different contributions to the co-propagation, while the propagation mechanism of *S*, $I_{i}$, $I_{j}$ and *I* are consistent, which *S* always reduces, $I_{i}$ and $I_{j}$ always increase firstly then decrease, and *I* always goes steadily up.

**Table 4:** Parameter results of two public hot events in Table 2.

Name	Estimated value	Standard error	Min	Max
$S_0$	8.965 $\times \;10^{5}$	82.6655	1.000 $\times \;\;10^{5}$	1.000 $\times \;10^{7}$
$\beta _i$	1.321 $\times \;10^{-4}$	1.002 $\times \;10^{-5}$	0.0000	0.0010
$\beta _j$	3.660 $\times \;10^{-5}$	2.102 $\times \;10^{-6}$	0.0000	0.0010
$p_i$	0.005	4.034 $\times \;10^{-4}$	0.0000	0.1000
$p_j$	0.013	9.586 $\times \;10^{-4}$	0.0000	0.1000
$\alpha _i$	0.105	0.0120	0.0000	2.0000
$\alpha _j$	0.388	0.0577	0.0000	2.0000
$\alpha _{ij}$	0.169	0.0201	0.0000	2.0000
$\alpha _{ji}$	1.293	0.0698	0.0000	2.0000
$m_{ij}$	0.174	0.0522	0.0000	2.0000
$m_{\bar{i}j}$	0.759	0.1012	0.0000	2.0000
$m_{\bar{j}i}$	0.565	0.0709	0.0000	2.0000
$m_{ji}$	1.094	0.0770	0.0000	2.0000

**Table 5:** Parameter results of two public hot events in Table 3.

Name	Estimated value	Standard error	Min	Max
$S_0$	1.016 $\times \;10^{5}$	96.6439	1.000 $\times \;10^{5}$	1.000 $\times \;10^{7}$
$\beta _i$	4.718 $\times \;10^{-5}$	7.038 $\times \;10^{-6}$	0.0000	0.0010
$\beta _j$	5.423$\times \;10^{-5}$	1.166 $\times \;10^{-6}$	0.0000	0.0010
$p_i$	0.050	0.0046	0.0000	0.1000
$p_j$	0.339	0.0697	0.0000	0.1000
$\alpha _i$	0.160	0.0362	0.0000	2.0000
$\alpha _j$	1.844	0.1361	0.0000	2.0000
$\alpha _{ij}$	0.247	0.0879	0.0000	2.0000
$\alpha _{ji}$	0.020	0.0197	0.0000	2.0000
$m_{ij}$	0.793	0.2656	0.0000	2.0000
$m_{\bar{i}j}$	0.262	0.0706	0.0000	2.0000
$m_{\bar{j}i}$	0.903	0.1706	0.0000	2.0000
$m_{ji}$	0.007	0.0072	0.0000	2.0000

**Table 6:** Public opinion indices of two public hot events in Tables 2 and 3.

	$\mathfrak {R}_0$	$C_s$	$F_{max}$	$t_d(10\;{\text {min}})$	$t_{max}(10\;{\text {min}})$	$V_b(/10\;{\text {min}})$	$V_d(/10\;{\text {min}})$
Event 1	6.1641	$1.2156\times 10^{4}$	$2.8949\times 10^{3}$	54.0099	9.7700	266.4025	58.9055
Event 2	1.4850	$1.9872\times 10^{4}$	$1.2261\times 10^{3}$	86.9416	31.6600	34.9190	19.9403

From data fitting, more public opinion properties can also be obtained. Tables 4 and 5 give the results of estimated parameters about the influencing factors to the co-propagation and cross-transmission of two pieces of information. The average exposure rate $\beta$ in information co-propagation is determined by different follow structures, $\beta _i=1.321\times 10^{-4}$ and $\beta _j=3.660\times 10^{-5}$ of event 1 have a large gap caused by such disparities of fans group, to the contrary, $\beta _i=5.423\times 10^{-4}$ and $\beta _j=4.718\times 10^{-4}$ of event 2 have a corresponding quantity. Due to the different attraction of different events, the probabilities $p_i=0.005$, $p_j=0.013$ of event 1 and $p_i=0.050$, $p_j=0.160$ of event 2 which represent the interest of each user to forward the information will keep stable within a certain range, with little difference in value varied by the content of information. Parameter $\alpha _i (\alpha _j)$ is the average immune rate of the information, and $1/\alpha _i (1/\alpha _j)$ is the average duration in which a normal forwarding user remains active in forwarding. Besides, parameter $\alpha _{ij} (\alpha _{ji})$ is the average immune rate of the information from a state in which one information is already immune to another information, and $1/\alpha _{ij} (1/\alpha _{ji})$ is the average duration in which a continuous attractive cross-transmission user remains active in forwarding. In our simulations, $\alpha _i=0.105$, $\alpha _j=0.388$, $\alpha _{ij}= 0.169$, $\alpha _{ji}=1.293$ of event 1 and $\alpha _i=0.160$, $\alpha _j=1.844$, $\alpha _{ij}= 0.247$, $\alpha _{ji}=0.020$ of event 2, the results show that $\alpha _i$, $\alpha _j$ determine the active ability of most forwarding behaviors and $\alpha _{ij}$, $\alpha _{ji}$ also control the state of existing immune to one information. And $1/\alpha _i$, $1/\alpha _j$, $1/\alpha _{ij}$ and $1/\alpha _{ji}$ are usually in the range [0.5,200] performed by our estimation. Most importantly, the strong attractive index $m_{ij}=0.174$, $m_{ji}=1.094$ and the continuous attractive index $m_{\bar{j}i}=0.565$, $m_{\bar{i}j}=0.759$ of event 1 and the strong attractive index $m_{ij}=0.793$, $m_{ji}=0.007$ and the continuous attractive index $m_{\bar{j}i}=0.903$, $m_{\bar{i}j}=0.262$ of event 2 indicate that the cross-transmission have various degree of impact on the co-propagation. And in event 1, the process with strong attractive accounts for a large proportion from information *j* to information *i*, and to the contrary, the process accounts for the smallest proportion in event 2. In addition, Tables 4 and 5 also give the relevant boundary conditions and the standard error for the parameter estimation of the two cases, which are all within acceptable range.

Table 6 gives the relative seven key public opinion indices that reveal the levels and patterns of co-propagation. Event 1 has the greatest reproduction ratio $\mathfrak {R}_0=6.1651$, and hence it approaches to $t_{max}$ = 9.7700 (10 min) and reaches the instantaneous maximum $F_{max}=2.8949\times 10^{3}$ quickly. Compared with event 1, event 2 reached to the instantaneous maximum $F_{max}=1.2261\times 10^{3}$ later which $t_{max}$ = 31.6600 (10 min) caused by a smaller reproduction ratio $\mathfrak {R}_0=1.4851$ and lasted longer than event 1. The cumulative number of forwarding users will not increase much based on the initial values and reach stability quickly. By calculating the life duration, the outbreak velocity and the declining velocity, $V_o$ = 266.4025 (/10 min) and $V_d$ = 58.9055 (/10 min) of event 1 is faster than $V_o$ = 34.9190 (/10 min) and $V_d$ = 19.9403 (/10 min) of event 2, and $t_d$ = 54.0099 (10 min) of event 1 is shorter than $t_d$ = 86.9416 (10 min) of event 2. The results indicate that two related information of event 1 brings so much attention from the information post owner’s followers or other audiences.

## Cross-transmission analysis

**Figure 13:**
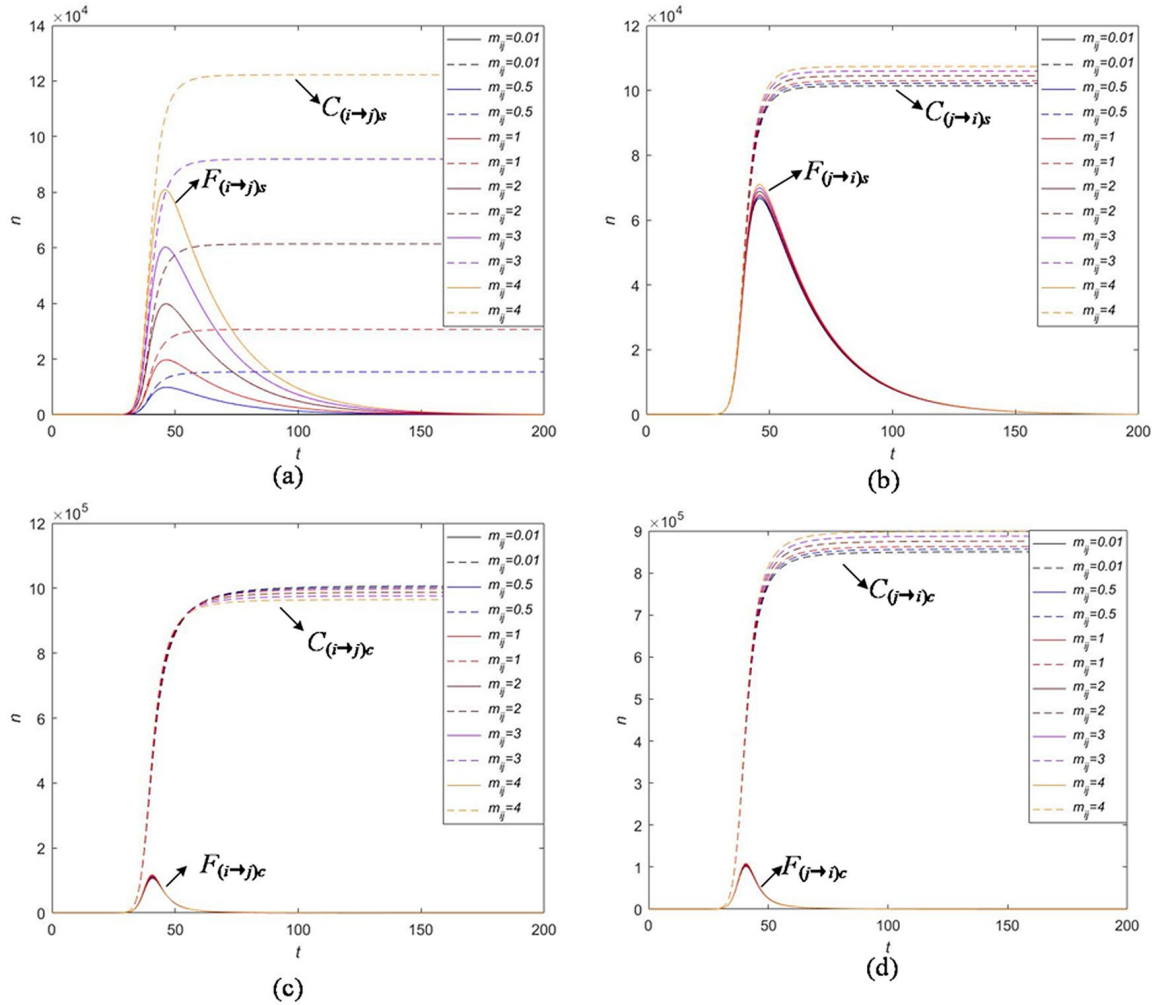
Numerical experiments on the effect of parameter $m_{ij}$ on cross-transmission with strong attraction and continuous attraction: (**a**) strong attraction from information *i* to information *j*; (**b**) strong attraction from information *j* to information *i*; (**c**) continuous attraction from information *i* to information *j*; (**d**) continuous attraction from information *j* to information *i*.

**Figure 14:**
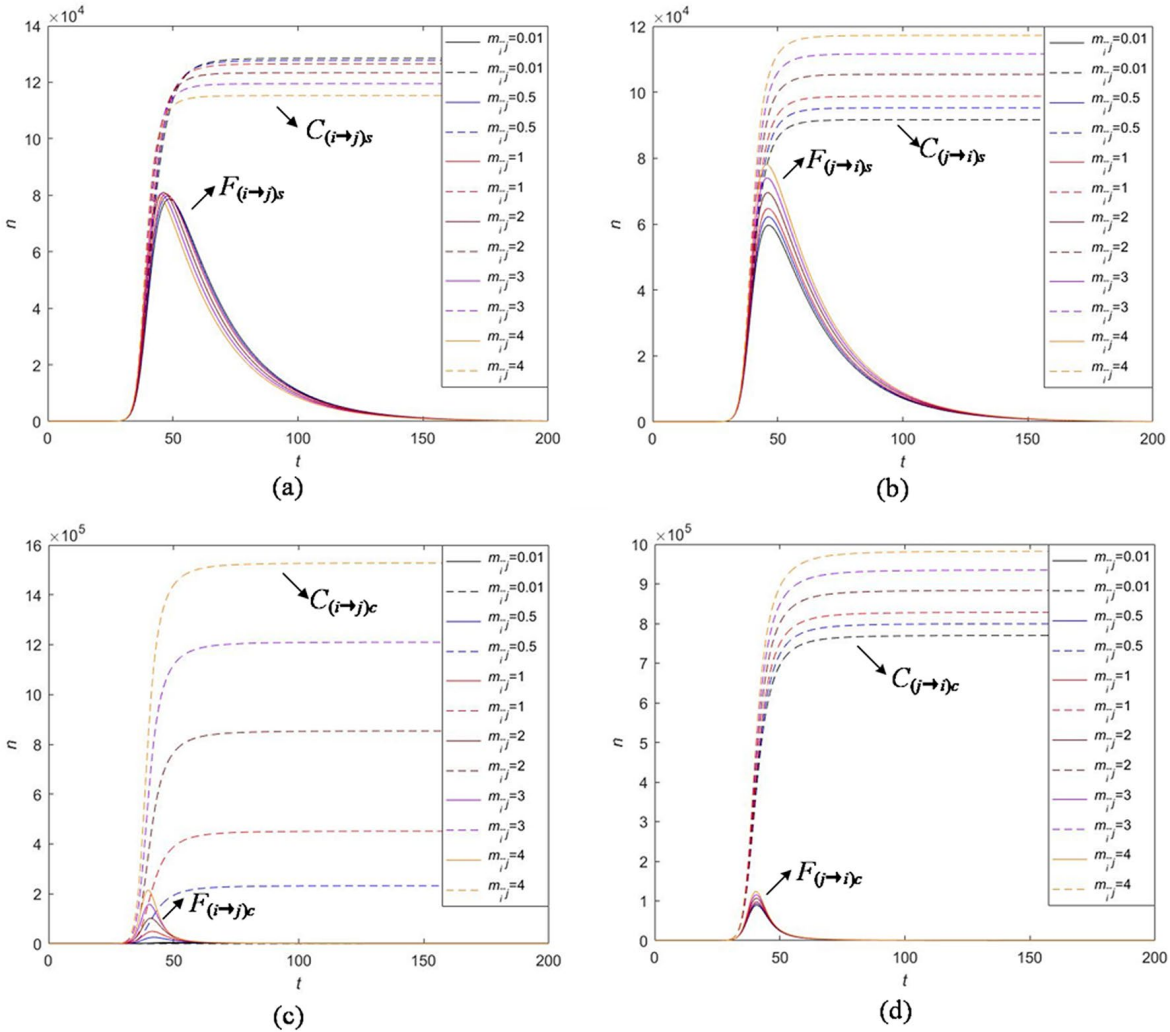
Numerical experiments on the effect of parameter $m_{\bar{i}j}$ on cross-transmission with strong attraction and continuous attraction: (**a**) strong attraction from information *i* to information *j*; (**b**) strong attraction from information *j* to information *i*; (**c**) continuous attraction from information *i* to information *j*; (**d**) continuous attraction from information *j* to information *i*.

The classical information propagation model only considers the exposure rate, forwarding probability and the immune rate to solve the practical problems. Our model concentrates on the influence of the strong and continuous attractive indexes between related information on the overall cross-transmission. Cross-transmission is the focus of our model and also an important factor affecting the co-propagation. The attraction between two related information is denoted by the parameters $m_{ij}$ and $m_{\bar{i}j} (i,j=1,2,i\not =j)$, which changes in different attraction and determines the co-propagation of the whole event. The definition of $m_{ij}$ and $m_{\bar{i}j}$ is in "Preliminaries". Here, we explore the influence of $m_{ij}$ and $m_{\bar{i}j}$ on $C_{(i\rightarrow j)s}$, $F_{(i\rightarrow j)s}$, $C_{(i\rightarrow j)c}$, $F_{(i\rightarrow j)c}$, $C_{(j\rightarrow i)s}$, $F_{(j\rightarrow i)s}$, $C_{(j\rightarrow i)c}$, $F_{(j\rightarrow i)c}$ by performing the sensitivity analysis as shown in Figs. 13 and 14.

From Fig. 13, we find that parameter $m_{ij}$ has obvious impact on the strong attractive population $C_{(i\rightarrow j)s}$ and $F_{(i\rightarrow j)s}$ and weakly effects on the cross-transmission population influenced by the continuous attraction $C_{(i\rightarrow j)c}$, $F_{(i\rightarrow j)c}$ and the cross-transmission population from information *j* to information *i* $C_{(j\rightarrow i)s}$, $F_{(j\rightarrow i)s}$, $C_{(j\rightarrow i)c}$, $F_{(j\rightarrow i)c}$. More specifically, increasing the strong attractive index $m_{ij}$ leads to a significant increase in the cumulative cross-transmission users $C_{(i\rightarrow j)s}$ and the high peak of instantaneous cross-transmission users $F_{(i\rightarrow j)s}$. Therefore, by choosing different values of $m_{ij}$, we can control the change of cross-transmission population in a short time and hence promote the integrated co-propagation of public hot event. Some corresponding strategies on the selection of $m_{ij}$ will be discussed in the "Information co-propagation control strategy".

From Fig. 14, we obtain that parameter $m_{\bar{i}j}$ has a relatively large effect on the continuous attractive population $C_{(i\rightarrow j)c}$ and $F_{(i\rightarrow j)c}$ but it is not a key parameter to control the cross-transmission population influenced by the strong attraction $C_{(i\rightarrow j)s}$, $F_{(i\rightarrow j)s}$ and the cross-transmission population from information *j* to information *i* $C_{(j\rightarrow i)s}$, $F_{(j\rightarrow i)s}$, $C_{(j\rightarrow i)c}$, $F_{(j\rightarrow i)c}$. More specifically, increasing the continuous attractive index $m_{\bar{i}j}$ is accompanied by the increases of cumulative cross-transmission users with continuous attraction from information *i* to information *j* and the high peak of instantaneous cross-transmission users $F_{(i\rightarrow j)c}$. In addition, $m_{ij}$ and $m_{\bar{i}j}$ influence the cross-transmission behavior in different degrees, and $m_{\bar{i}j}$ has more obvious effect. That is, it is more effective to control $m_{\bar{i}j}$ to promote the outbreak of information and expand the co-propagation.

To sum up, for $m_{ij}$, more users will forward information *j* with strong interest within a short time as $m_{ij}$ goes up, but the surviving period of the whole event has no substantial changes; The increase of $m_{\bar{i}j}$ leads to the increase of the beginning and declining velocities of co-propagation and has no effect on the surviving period. The results provide guidelines for controlling the cross-transmission users who have become immune or inactive to the information *i*. That is to say, when we attempt to expand propagation, whereas two related information has different heats on the event, the information attracts the users who are willing to support and forward additional information about this event. Hence, one main contribution of this paper is that we analyze the features of cross-transmission created by susceptible users’ interests on the relation information of some public hot events.

## Co-propagation analysis

To further analyze the different parameters responsible for the integrated co-propagation, the PRCCs (Partial Rank Correlation Coefficient)^31^ is performed to study the influences to the cumulative forwarding population and variation of cross-transmission population, where the value of different parameters are inputted for the sensitivity analysis of 1000 samples under the specific threshold. According to the histogram and scatter diagram of $\mathfrak {R}_0^1$ dependence, when the PRCC value is positive (negative), the corresponding index will increase (decrease) as the parameter increases (decreases).

**Figure 15:**
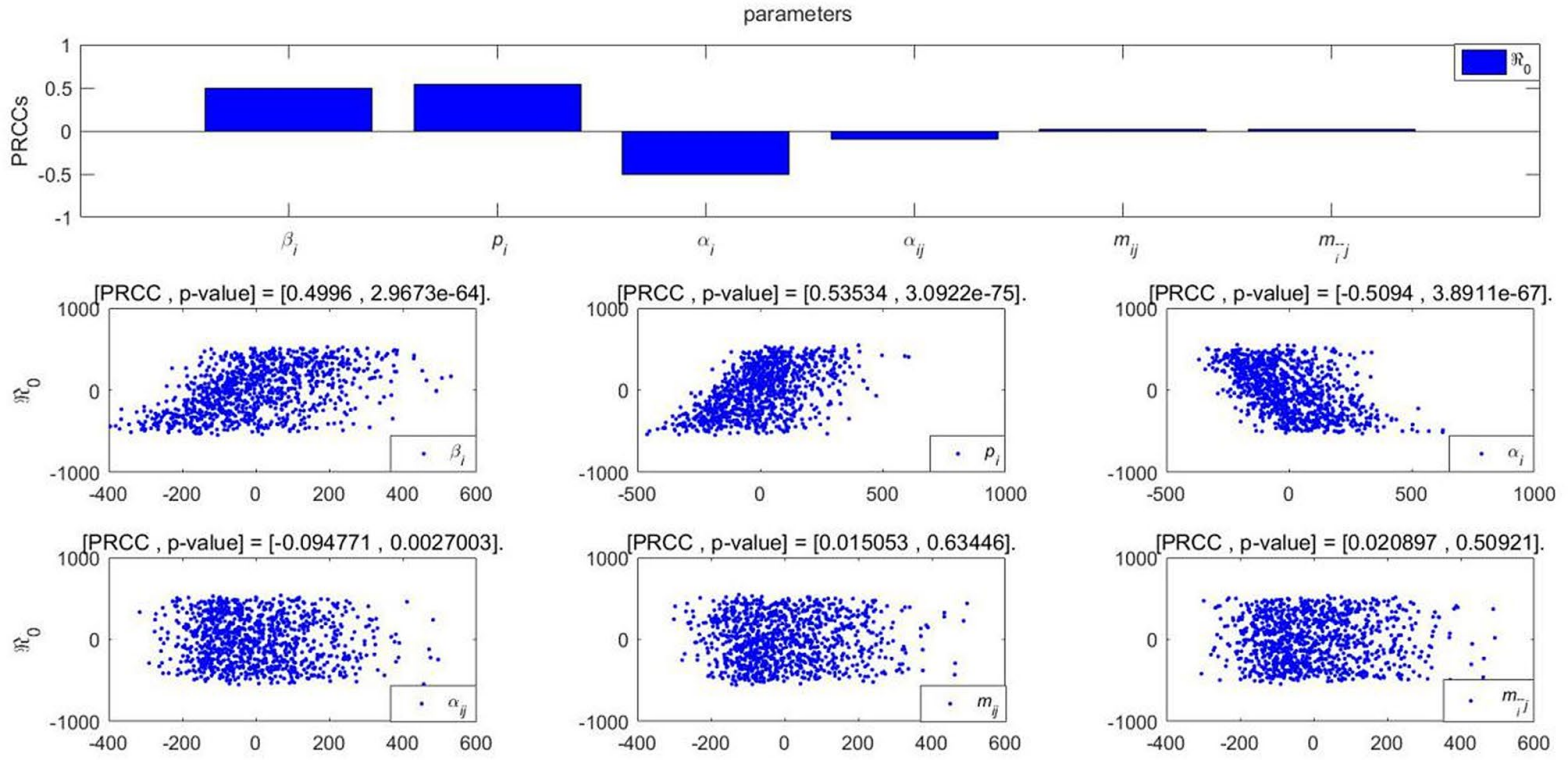
PRCC results and PRCC scatter plots with index $\mathfrak {R}_0$ of different parameters.

**Figure 16:**
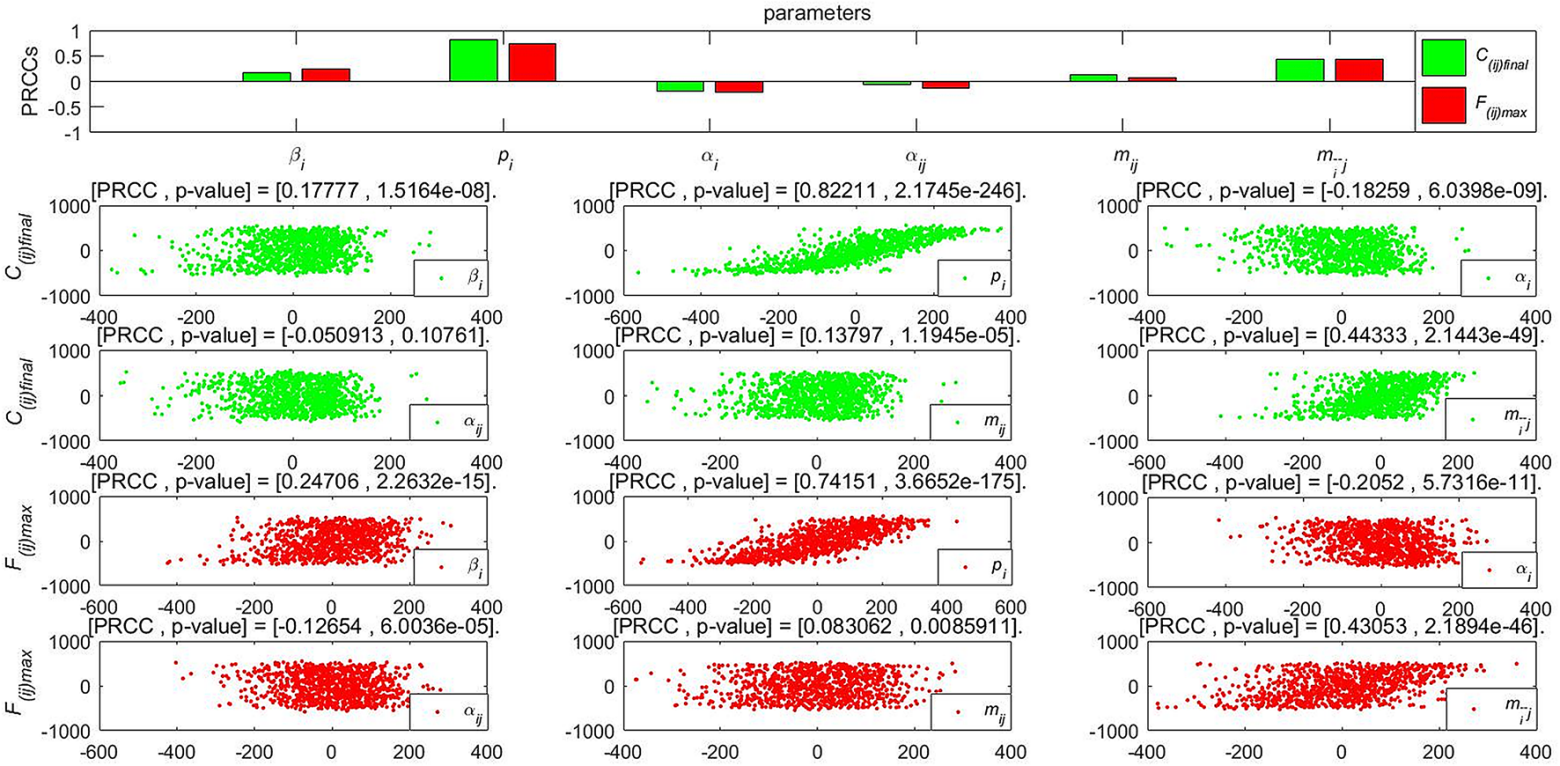
PRCC results and PRCC scatter plots with population $C_{( ij)final}$ and $F_{( ij)max}$ of different parameters.

**Figure 17:**
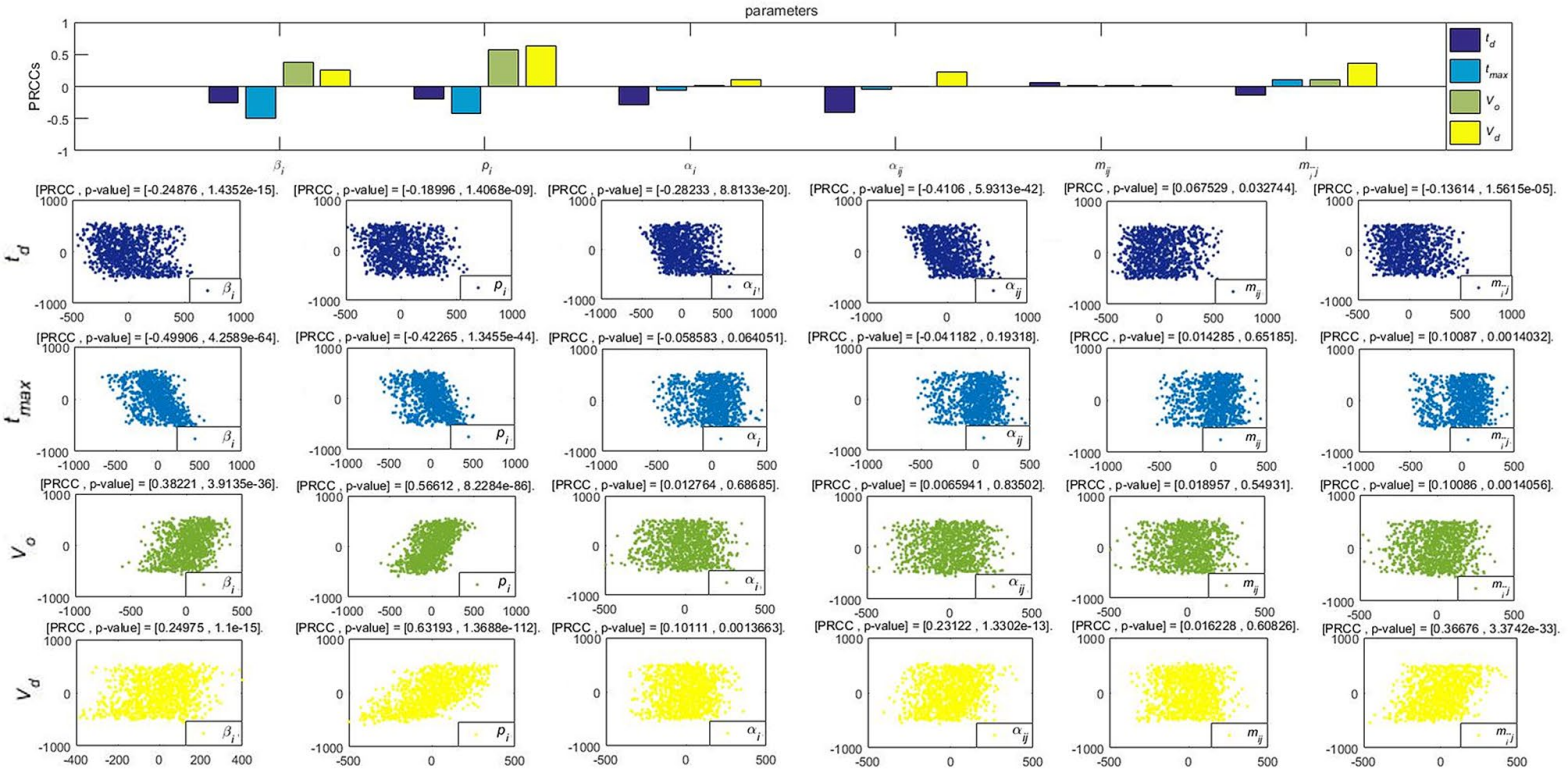
PRCC results and PRCC scatter plots with indexes $t_d$, $t_{max}$, $V_o$ and $V_d$ of different parameters.

Figures 15, 16 and 17 give the PRCC results and PRCC scatter plots with seven key indices in public opinion event mentioned above ($\mathfrak {R}_{0}$, $C_{( ij)final}$, $F_{( ij)max}$, $t_{d}$, $t_{max}$, $V_{o}$, $V_{d}$) with different parameters (take $\beta _{i}$, $p_{i}$, $\alpha _{i}$ $\alpha _{ij}$, $m_{ij}$, $m_{\bar{i}j}$ for example), respectively. We choose 0.4 as the threshold, when the absolute value |PRCC| $\ge$0.4, it is considered that the parameter has a strong correlation effect on the indice, that is to say, the indice can be effectively controlled by taking strategies on this parameter, when the absolute value 0.2$\le |$PRCC$|<0.4$, it is considered that this parameter has a median influence on the value of the indice, and when the absolute value |PRCC$|<0.2$, the parameter works a weak correlation.

We know from For Fig. 15 that $\beta _{i}$, $p_{i}$, $\alpha _{i}$ are critical parameters to influence the increase of the values of $\mathfrak {R}_{0}$ in comparison with parameters $\alpha _{ij}$, $m_{ij}$, $m_{\bar{i}j}$. This result confirms the correctness of our derivation of $\mathfrak {R}_{0}$ in Eqs. (22). Therefore, the average contacted rate $\beta _{i}$, the forwarding probability $p_{i}$ and the average immune rate $\alpha _{i}$ are the key factors to determine the outbreak of the event.

Figure 16 shows the effect of parameters on the values of integrated co-propagation. In this part, we focus on how the cross-transmission of two related information influence the co-propagation. Compared with other parameters, $p_{i}$ or $m_{\bar{i}j}$ have a strong influence on the integrated co-propagation of instantaneous forwarding users $F_{( ij)max}$ and deeper influence on cumulative forwarding users $C_{( ij)final}$ intuitively. For this reason, increasing the average forwarding probability $p_{i}$ or continuous attractive index $m_{\bar{i}j}$ is useful to broaden the co-propagation and reach its high peak of co-propagation quickly. This means that the proposed attractive indexes are vital parameters to promote the cross-transmission and hence the co-propagation of information. In the real network, different cross-transmission of the event may generate different influences and consequences of co-propagation.

Figure 17 depicts the effect of parameters on the values of some vital times and velocities. For the duration $t_d$, parameter $\alpha _{ij}$ make strong contributions to decrease it, $\beta _{i}$ and $\alpha _{i}$ play a medium role in $t_d$. For the time of high peak $t_{max}$, $\beta _{i}$ and $p_{i}$ are the mainly parameters controlling its decrease. For the outbreak velocity $V_o$ and the decline velocity $V_d$, $p_i$ is the main control factor to determine the change of velocities. In addition, $\beta _{i}$ moderately contributes to the increase of the outbreak velocity $V_o$ and $\beta _{i}$, $\alpha _{ij}$, $m_{\bar{i}j}$ are medially contributes to the increase of decline velocity $V_d$. According to the results, the strong and continuous attractive indexes have a great effect on the final size and the high peak but has no significant effect on the integrated times and velocities.

## Information co-propagation control strategy

Nowadays there are several types of public opinion available: rumor, positive propagation needed or advertisements, two pieces of information: one positive and one negative on guidance of public opinion. Different coping strategies are needed for different types, we list them as:

When rumors spreading in the social network, we can limit the $C_{( ij)final}$ and $F_{( ij)max}$ of integrated event by reducing the forwarding probability $p_{i}$ ($p_{j}$), continuous attractive index $m_{\bar{i}j}$ ($m_{\bar{j}i}$) and increasing average immune rate $\alpha$. To realize this, the government can take some effective measures, including improving the media credibility, enhancing public awareness and education, and strengthening the monitoring of public opinion.

When positive propagation or advertisements need to be widely disseminated in social networks, we can amplify the capacity of co-propagation $C_{( ij)final}$ and $F_{( ij)max}$ by increasing parameters $m_{\bar{i}j}$ ($m_{\bar{j}i}$) and $p_{i}$ ($p_{j}$). Because $p_{i}$ ($p_{j}$) is the forwarding probability for a user to transmit the information with some interest, and $m_{\bar{i}j}$ ($m_{\bar{j}i}$) is the continuous attractive index for the cross-transmission which contributes to the expansion of integrated co-propagation. The operation of parameter control can be targeted to provide strategies. For example, try to make the post owners who post information *i* and information *j* to be opinion leaders with large fan groups, and two opinion leaders simultaneously post different parts of the event contents or from different perspectives, to create a mutually influential relationship and give rise to the strong or continuous attraction between the two major fan groups and enable users to forward information one after another subsequently. In conclusion, try to enrich the content of information to increase the user’s interest, which will improve the probability *p* and the attractive index $m_{\bar{i}j}$ ($m_{\bar{j}i}$) of users who forwarding information. When a negative piece of information appears in an event, it may stir up negative emotions (here, we assume information *i* is the positive one and information *j* is the negative one). We hope other positive information can spread more widely and the negative impact of information is minimized. This can be achieved by increasing the probability $p_i$, the strong attractive index $m_{ji}$ and continuous attractive index $m_{\bar{j}i}$. It would be useful to persuade more opinion leaders to forward the positive information and attach more favorable views, to attract susceptible users and other users who have forwarded or were immune to the information of another opinion to the positive information. Conversely, we can reduce the focus of the negative information by decreasing $p_{j}$, $m_{ij}$, $m_{{\bar{j}}i}$, $m_{{\bar{i}}j}$ and increasing the discussion of positive information through opinion leaders.

## Conclusion and discussion

In this paper, we proposed a synchronous information cross-transmission dynamics CT-SFI model to understand the diffusion pattern of two related information spreading on the internet. Since there are always two or more kinds of information spreading simultaneously in the real world, analyzing the cross-transmission mechanism between them is very necessary and significant. For two related information posted in the same time period, we considered the cross-transmission between them and studied their co-propagation dynamic process, where the strong attractive index $m_{ij}$ ($m_{ji}$) and the continuous attractive index $m_{\bar{i}j}$ ($m_{\bar{j}i}$) are proposed to describe the cross-transmission. The core of our model is to explore the cross-transmission behavior generated by users by investigating attractive indexes and to effectively control the co-propagation of the public hot event by designing some strategies.

By performing the data fitting, we carried out numerical simulations to verify the effectiveness of the CT-SFI model and studied the influence of the strong attractive index $m_{ij}$ ($m_{ji}$) and the continuous attractive index $m_{\bar{i}j}$ on some important cross-transmission indices $C_{(i\rightarrow j)s}$, $F_{(i\rightarrow j)s}$, $C_{(i\rightarrow j)c}$, $F_{(i\rightarrow j)c}$, $C_{(j\rightarrow i)s}$, $F_{(j\rightarrow i)s}$, $C_{(j\rightarrow i)c}$, $F_{(j\rightarrow i)c}$. The simulation results illustrate the influences of different attractive indexes on the cumulative forwarding number $C_{(i\rightarrow j)s}$ ($C_{(j\rightarrow i)s}$) of strong attractive cross-transmission users within an active forwarding period and the cumulative forwarding number $C_{(i\rightarrow j)c}$ ($C_{(j\rightarrow i)c}$) of continuous attractive cross-transmission users within an insensitive period, and $m_{ij}$, $m_{\bar{i}j}$ are characterization factors of the cross-transmission: the greater values of $m_{ij}$ and $m_{\bar{i}j}$, the more attraction between two related information and the greater $C_{(i\rightarrow j)s}$ and $C_{(i\rightarrow j)c}$. We provided the PRCC experiment of seven key indices in public opinion event ($\mathfrak {R}_{0}$, $C_{( ij)final}$, $F_{( ij)max}$, $t_{d}$, $t_{max}$, $V_{o}$, $V_{d}$) with different parameters (take $\beta _{i}$, $p_{i}$, $\alpha _{i}$ $\alpha _{ij}$, $m_{ij}$, $m_{\bar{i}j}$ for example) to analyze the impact of cross-transmission on the co-propagation of information. The PRCC results indicate that the cross-transmission with continuous attractive index has a more obvious impact on the co-propagation than that with strong attractive index, and the key influencing factors of co-propagation include the forwarding probability $p_{i}$ ($p_{j}$), the continuous attractive index $m_{\bar{i}j}$ ($m_{\bar{j}i}$) and the average immune rate $\alpha _{i}$ ($\alpha _{j}$). Hence, the cross-transmission conduces to the final size and the high peak of integrated co-propagation. Based on our conclusions, some strategies are proposed for different types of information.

The mutual attraction of information studied in the paper is of great practical importance since the cross-transmission between two related information plays a vital role in the design of efficient strategies to control information spreading. The proposed CT-SFI model breaks the restriction of many previous works that only considered the single information spreading patterns, and this breakthrough makes the CT-SFI model more realistic in the interaction of information. The methods in this paper is the basis for the studies of multiple information propagation in the real network.
